# Eukaryotic and cyanobacterial communities associated with marine snow particles in the oligotrophic Sargasso Sea

**DOI:** 10.1038/s41598-019-45146-7

**Published:** 2019-06-20

**Authors:** Regitze B. C. Lundgreen, Cornelia Jaspers, Sachia J. Traving, Daniel J. Ayala, Fabien Lombard, Hans-Peter Grossart, Torkel G. Nielsen, Peter Munk, Lasse Riemann

**Affiliations:** 1 Marine Biological Section, Department of Biology, University of Copenhagen, Helsingør, Denmark; 2 GEOMAR Helmholtz Centre for Ocean Research Kiel, Evolutionary Ecology of Marine Fishes, Kiel, Germany; 3 Present Address: National Institute of Aquatic Resources, Technical University of Denmark, Kgs. Lyngby, Denmark; 4 Sorbonne Université, CNRS-INSU, Laboratoire d’Océanographie de Villefranche, LOV UMR 7093, F-06230 Villefranche-sur-mer, France; 5 Leibniz-Institute of Freshwater Ecology and Inland Fisheries (IGB) Berlin, Stechlin, Germany; 6 Potsdam University, Institute for Biochemistry and Biology, Potsdam, Germany; 7 Present Address: Department of Microbiology and Immunology, University of British Columbia, Vancouver, Canada

**Keywords:** Ecosystem ecology, Marine biology

## Abstract

Marine snow aggregates represent heterogeneous agglomerates of dead and living organic matter. Composition is decisive for their sinking rates, and thereby for carbon flux to the deep sea. For oligotrophic oceans, information on aggregate composition is particularly sparse. To address this, the taxonomic composition of aggregates collected from the subtropical and oligotrophic Sargasso Sea (Atlantic Ocean) was characterized by 16S and 18S rRNA gene sequencing. Taxonomy assignment was aided by a collection of the contemporary plankton community consisting of 75 morphologically and genetically identified plankton specimens. The diverse rRNA gene reads of marine snow aggregates, not considering *Trichodesmium* puffs, were dominated by copepods (52%), cnidarians (21%), radiolarians (11%), and alveolates (8%), with sporadic contributions by cyanobacteria, suggesting a different aggregate composition than in eutrophic regions. Composition linked significantly with sampling location but not to any measured environmental parameters or plankton biomass composition. Nevertheless, indicator and network analyses identified key roles of a few rare taxa. This points to complex regulation of aggregate composition, conceivably affected by the environment and plankton characteristics. The extent to which this has implications for particle densities, and consequently for sinking rates and carbon sequestration in oligotrophic waters, needs further interrogation.

## Introduction

Sequestration of atmospheric carbon to the deep ocean is mainly mediated via sinking particulate organic matter (POM^1^). Macroscopic detrital aggregates >500 µm, also known as marine snow^2^, are usually formed by physical and chemical coagulation processes as well as biological processes including zooplankton activity^3,4^. Generally, they are highly abundant in the upper 2,000 m of the water column^5^. The sinking of marine snow has been estimated to remove up to 3–5% and 4–22% of the particulate organic carbon and nitrogen, respectively, from marine surface waters per day in Monterey Bay and the northeast Atlantic Ocean^6^. Sinking rate of particles, which in turn determines removal from surface waters, is highly variable (approx. 1–370 m d^−1^)^7–9^, and differs greatly between similar-sized particles^3,10–12^. Hence, sinking rate is dependent on particle density rather than size, and is thereby highly influenced by the taxonomic composition of the particle^3,12,13^.

While marine snow is often described as detritus^14^, as individual components are hard to discern morphologically, living organisms may also contribute to marine snow aggregates^3,4,15–17^. So far, most studies of marine snow composition have concerned rather eutrophic marine regions, revealing a predominance of diatom flocs, dinoflagellates, coccolithophorids, appendicularian (larvacean) houses, and fecal aggregates, but also amorphous particles of unknown origin^10,18–22^. In particular, diatoms appear to be common constituents of marine snow^20,23,24^ and are considered major contributors to the vertical carbon flux in eutrophic regions^25–28^, although appendicularians may also be important^20,29–31^.

In comparison, although particulate matter is found from the surface to the bathypelagic of the oligotrophic oceans^32^, information on marine snow composition in these regions is sparse, apart from well-studied long term stations covered by the BATS (Atlantic) and HOT (Pacific) programs^33,34^. Whereas eutrophic regions generally support a classical food chain with large phyto- and zooplankton, oligotrophic stratified waters are often dominated by picoplankton^35^ and a relatively high heterotrophic biomass^36^, including extensive carbon cycling through the microbial loop^37^. Even though diatoms are rare and seldom reach high abundances in subtropical oligotrophic gyres^35,38,39^, diatom blooms have been shown to frequently occur in oligotrophic open-ocean waters, e.g. at the Atlantic time series station BATS^33^. Irrespectively, diatoms are not expected to contribute significantly to marine snow formation on an annual basis^33^. Instead, filter feeding organisms, like appendicularians which are specialized in picoplankton grazing^29^, are expected to be of higher importance^40^. As an example, discarded appendicularian houses contributed up to 60% of the particulate organic carbon flux in the oligotrophic Ligurian Sea (NW Mediterranean Sea)^41^. Consequently, in oligotrophic waters with a predominance of low-density gelatinous taxa^42^, particle density, and thereby sinking velocity, would likely differ from eutrophic waters where particles are dominated by heavier diatom or copepod faecal pellets^12,25^. Hence, insight into particle composition is important for our understanding of aggregate features and therefore, the vertical carbon flux, yet our knowledge is rather limited for oligotrophic waters in general.

Considering the fundamental differences between food web constituents of eutrophic and oligotrophic waters, large differences in the composition of marine snow would be anticipated, but this has so far not been investigated based on molecular characterization of individual particles. Hence, it remains unknown to what extent soft-bodied, transparent, or fast degrading food web components contribute to the marine snow particle composition and affect their sinking rates. Two major questions emerge: (1) whether the relationship between aggregate features and nutrient status of the system is changing, and (2) whether marine snow composition reflects the planktonic species community composition. Previous studies of marine snow composition have often relied on microscopical identification, which puts emphasis on larger components such as copepod and phytoplankton remains^43,44^. Recent technological advances now allow for image analyses of marine snow particles from underwater vision profilers, though the identification of those particles is dependent on image quality^32^. Thus, identification of non-characteristic or degraded particles remains problematic. Molecular analyses may offer an advantage over traditional methods as even heavily degraded organismic remains can be detected and identified due to the usage of short DNA amplicons^45^, although the outcome can be affected by DNA degradation or inefficient DNA extraction from some organisms, primer selectivity causing preferential amplification of targets, as well as differences in copy number per genome between taxa^45–48^. Hence, 18S rRNA gene metabarcoding is increasingly used for plankton biodiversity assessment^49–51^ although the approach should be considered only semi-quantitative^52^. Nevertheless, such analyses can move beyond the limits set by morphological identification. Moreover, if assuming that the extracted DNA originates from the organisms constituting the marine snow, then the metabarcoding will allow for a more detailed and precise identification of constituents in marine snow particles.

Therefore, in the present study, we examined the composition of marine snow particles from the oligotrophic Sargasso Sea located in the subtropical North Western Atlantic Ocean. Individual marine snow particles (excluding *Trichodesmium* aggregates) were obtained from stations differing in hydrography and plankton community composition and productivity, and aggregate composition was analysed by simultaneous 18S and 16S rRNA gene PCR amplicon sequencing. The following hypotheses were tested: (a) the composition of marine snow in the oligotrophic Sargasso Sea differs from eutrophic regions, and (b) marine snow composition differs between stations reflecting environmental variables at the respective stations.

## Results

**Figure 1:**
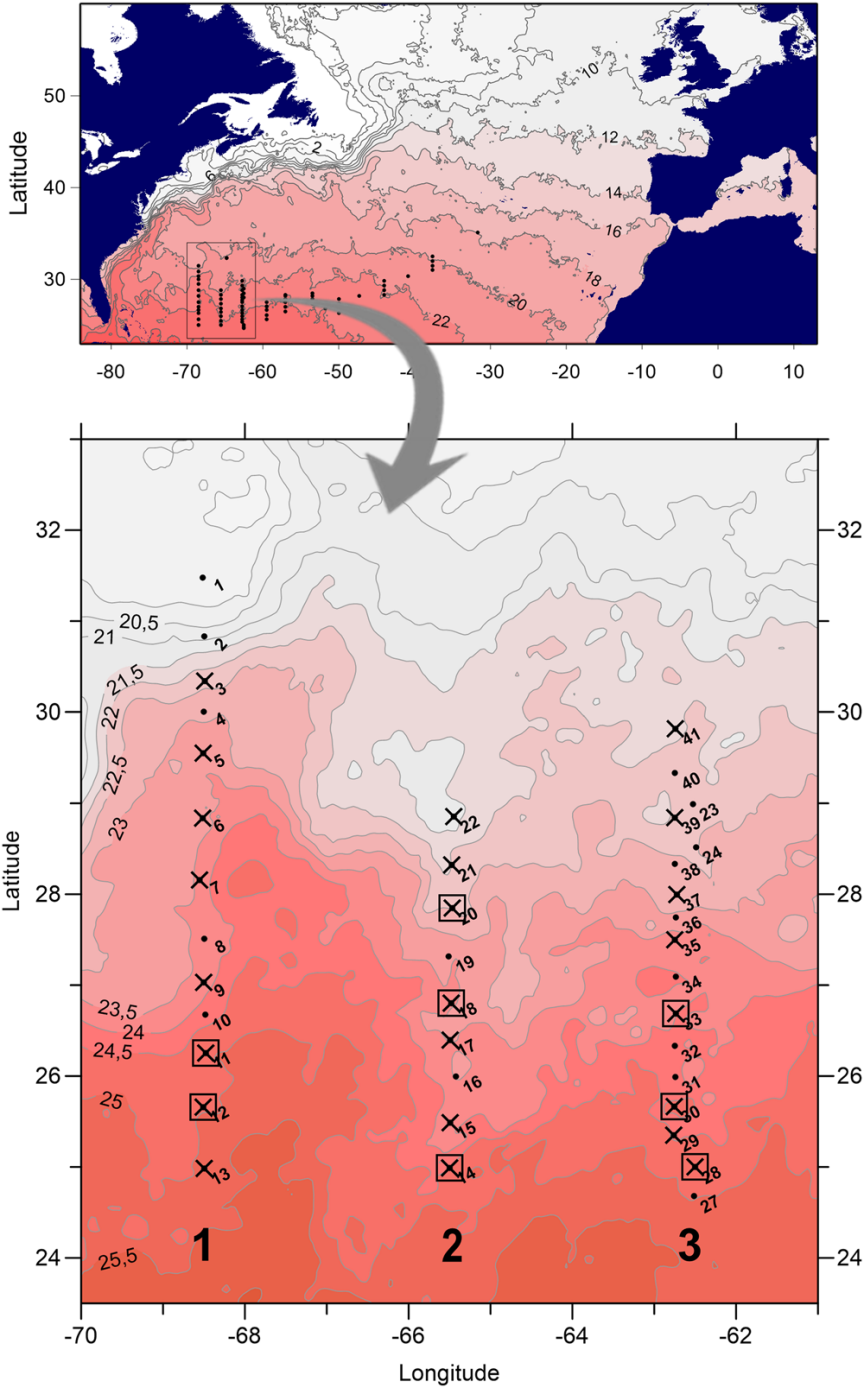
Maps of the Atlantic Ocean and Sargasso Sea showing transects and sampled stations. Isolines indicate surface temperature (°C) as determined from satellite images. Positions of CTD casts (black dots), of selected plankton sampling (crosses) and of snow sampling (squares) are indicated. Station numbers are labelled to the right of symbols. Large numbers at the bottom indicate transect numbers. The maps were made using Surfer® version 13.

Samples were collected during a cruise with R/V DANA (Technical University of Denmark) between 16^th^ of March and 5^th^ of April 2014, in the Sargasso Sea. A total of 41 stations were sampled along three north-south transects (Fig. 1). Of these, 22 stations were sampled for plankton and marine snow (Underwater Vision Profiler), and individual marine snow particles were obtained from 8 stations.

### Hydrography and plankton composition and distribution

**Figure 2:**
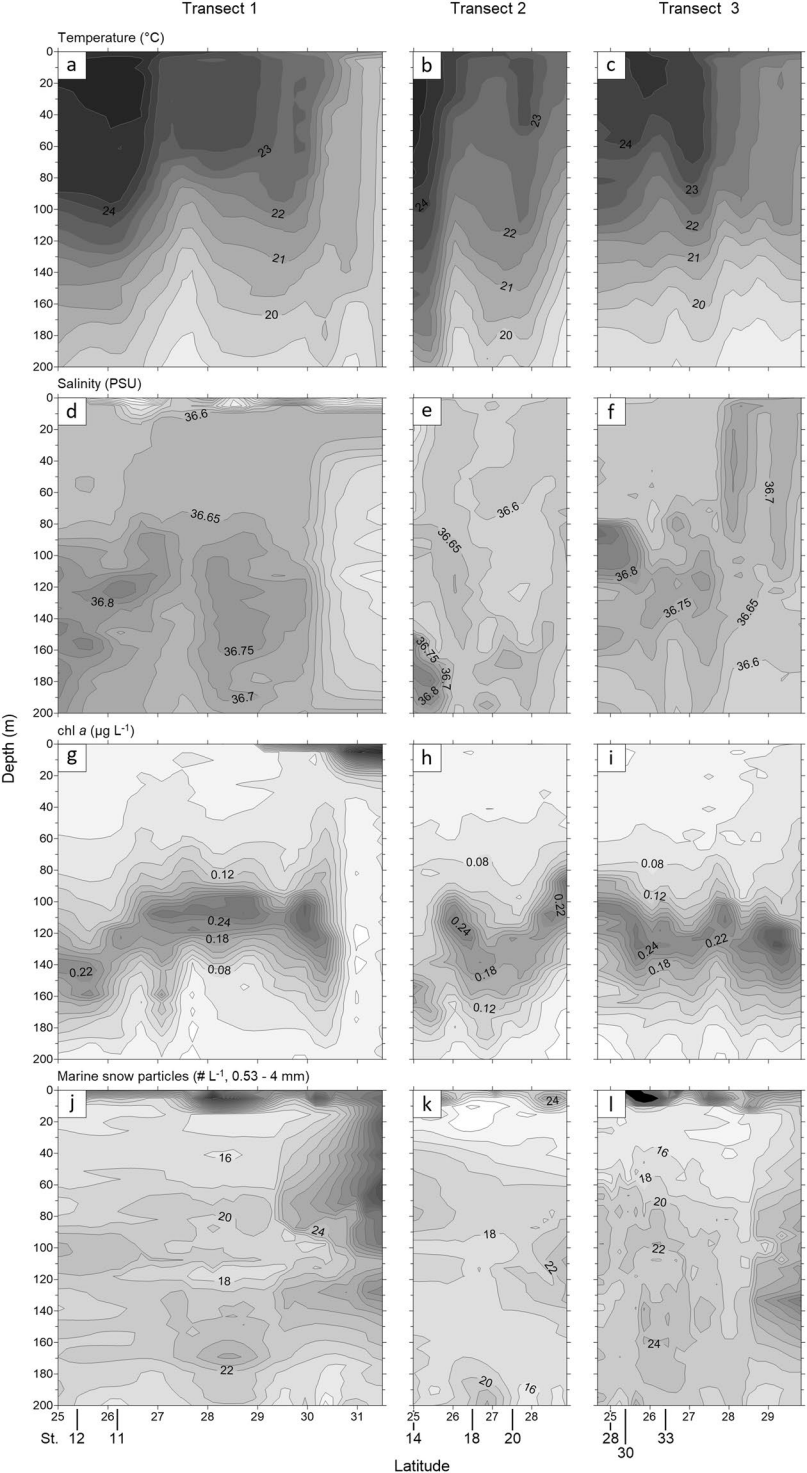
Temperature, salinity, chlorophyll *a*, and marine snow abundances (incl. *Trichodesmium* puffs) at Transect 1 (left column), Transect 2 (middle column), and Transect 3 (right column). ABC = Temperature (°C), DEF = salinity (PSU), GHI = chlorophyll *a* (µg L^−1^), JKL = marine snow abundances (# L^−1^, 0.53–4 mm). Stations sampled for marine snow are indicated on the latitude axis of JKL.

The stations sampled for marine snow encompassed differences in temperature, salinity, and phytoplankton biomass (chl *a*; Figs 1 and 2). At southern stations, the thermocline was evident at approx. 100–140 m depth, while a frontal zone was characterized by a lifting of the thermocline, and shallowing of the upper mixed layer to approx. 60 m. A frontal zone was present at 26–27 degrees latitude on Transects 1 and 2, while the front had moved north on Transect 3 (Fig. 2). In the frontal zone, warm water masses from the south (24–25 °C) met with colder waters from the north (20–22 °C; Fig. 2a–c). Salinity ranged from 32.98 to 37.0 (Fig. 2d–f). Except for the northern-most stations at Transect 1, the deep chl *a* maximum was associated with the thermocline (up to 0.42 µg chl *a* L^−1^, Fig. 2g–i). Moreover, abundances and depth distribution of marine snow particles (including *Trichodesmium* puffs) from the video plankton recorder showed differences between stations (Fig. 2j–l). For instance, marine snow particles on Transects 1 and 3 showed higher abundances in surface waters relative to Transect 2. Overall, marine snow particles were more abundant in the northern parts of the transects; in the southern parts, two abundance peaks were observed in the water column: in the surface layer and below the deep chl *a* maximum.

**Figure 3:**
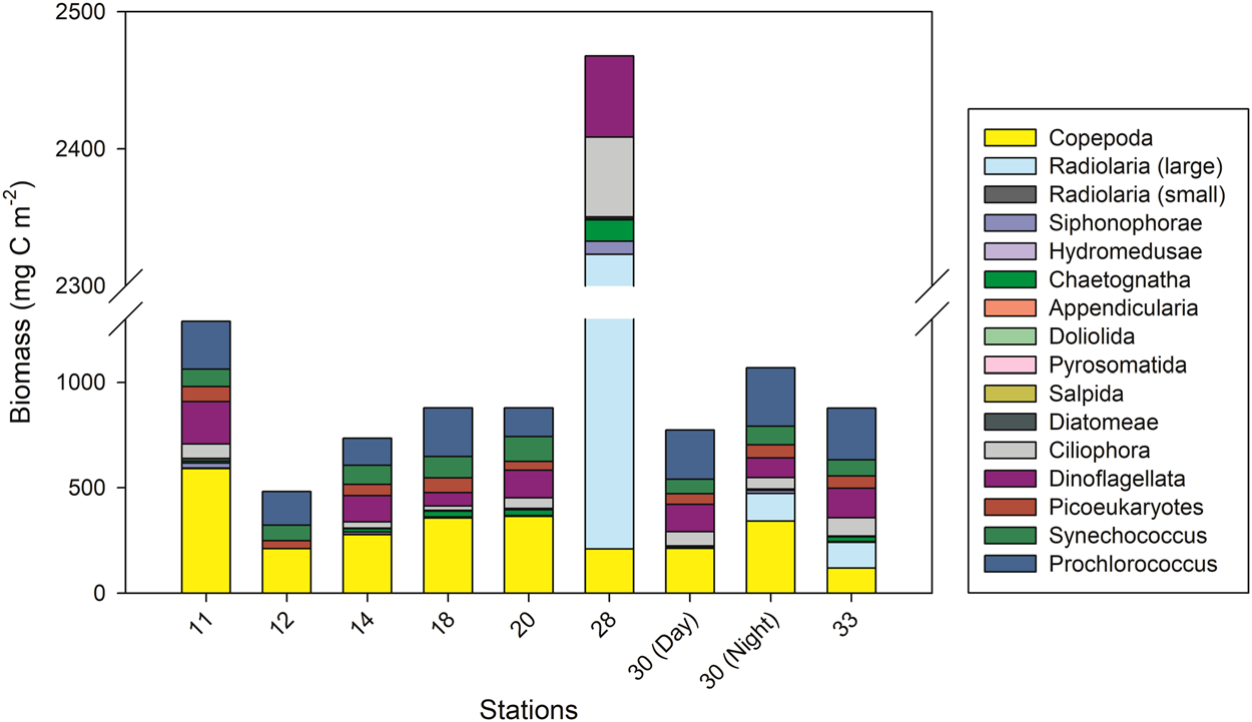
Biomass of the main plankton taxa at the stations sampled for marine snow. Data integrated to 200 m depth. Radiolarians are divided into small (av. size 0.15 mm) and large (av. size 5 mm) specimens. For details, see Table S1.

The biomass of the main metazoan and phytoplankton (diatoms, dinoflagellates, picoeukaryotes, *Synechococcus*, and *Prochlorococcus*) groups varied between the stations sampled for marine snow (Fig. 3, Table S1). Copepods dominated the metazoan biomass at most stations, followed by chaetognaths and siphonophores (Fig. 3; Table S1). The remaining metazoan groups showed a consistently low biomass in comparison. The biomass of heterotrophic dinoflagellates, ciliates, and cyanobacteria (excluding *Trichodesmium*) was higher than the metazoan biomass at most stations aside from copepods. Large radiolarians showed an extremely high biomass with 2 g C m^−2^ at one station (Station 28), and showed overall high biomasses when present. *Prochlorococcus* biomass was higher than that of *Synechococcus*, picoeukaryotes, and heterotrophic dinoflagellates, respectively (Table S1). Diatoms, though present at low biomass, were found at all stations, except south of the front at Station 11 on Transect 1.

### Composition of marine snow particles

**Figure 4:**
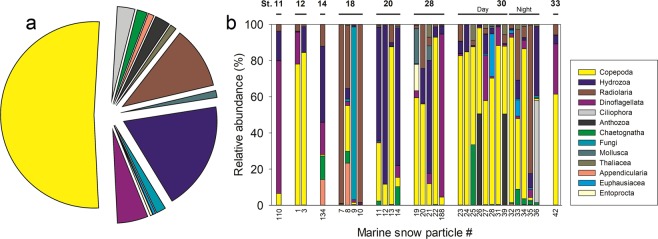
Marine snow composition based on 18S rRNA gene sequencing. (**a**) The average composition of 31 marine snow particles. (**b**) Composition of individual marine snow particles. For detailed information on specific particle numbers see Table S4. Particles are sorted by transects and stations. Note that station 30 is divided into day and one night sampling.

Based on 18S rRNA gene sequencing, a total of 826 OTUs were obtained from 31 successfully amplified marine snow samples with a size (length) of 1–10 mm. After removal of OTUs with <9 reads in total, 769 OTUs representing 411,444 reads remained. Fifty of the most dominant OTUs clustered in 13 taxonomic groups (Figs 4a and S1). Copepoda accounted for 18 of the dominant OTUs (Table S2) and 52% of the total number of reads (Fig. 4a). The most prevalent OTU (14% of reads; Table S2) was 98% similar to the copepod *Clausocalanus furcatus*. The next most prevalent OTU was identical to several unidentified calicophoran siphonophore species, including the commonly observed species *Abylopsis eschscholtzii* (11% of the reads), and the third most prevalent OTU was 99% similar to the copepod *Paracalanus parvus* (7% of total reads). Other dominant groups included cnidarians (21% of reads; mainly hydrozoans with 19% of reads), radiolarians (11% of reads), and alveolates (8% of reads; mainly dinoflagellates with 5% of reads). The sum of all remaining groups accounted for only 8% of the reads associated with the 50 most dominant OTUs.

Substantial variability in composition between individual marine snow particles was observed (Fig. 4b). The four main taxonomic groups were present in almost all marine snow particles but varied in relative abundance (average ± standard deviation; Copepoda: 44 ± 35%; Hydrozoa: 16 ± 24%; Radiolaria: 14 ± 28%; Dinoflagellata: 10 ± 21%). The remaining groups were found more sporadically.

Based on the 16S rRNA gene sequencing, cyanobacteria were present in most particles, i.e. they accounted for >0.5% of the gene reads in 90% of the marine snow particles, but the cyanobacterial contribution was highly variable with *Trichodesmium*, *Synechococcus*, and *Prochlorococcus* accounting for 13 $\pm$ 26%, 1.8 $\pm$ 8.4%, and 2.0 $\pm$ 6.8% of the reads per particle (average ± standard deviation, n = 31).

### Linkage of marine snow composition to measured physical and biological parameters

**Figure 5:**
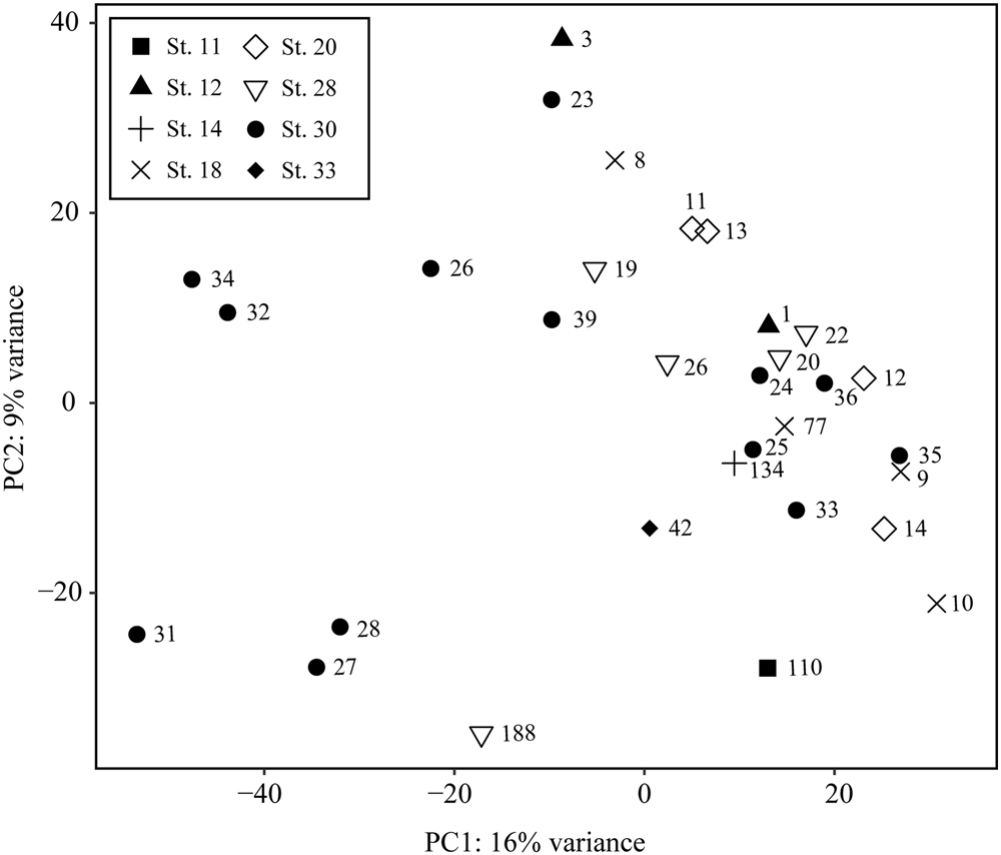
Principal component analysis (PCA) of the similarities in 18S rRNA gene composition of the marine snow particles, across stations, based on relative  read abundances. Symbols indicate sampling station, and numbers the snow particle #.

There was a significant effect of stations on marine snow particle composition (Generalized Linear Model (GLM), P = 0.012) suggesting that composition varied with sampling location. However, no specific OTUs came up as significantly contributing to this relationship between stations and composition. Also, no clear relationship was evident from the principal component analysis (Fig. 5), though it should be noted that the plotted components only resolve 25% of the total variation in the marine snow particles. Furthermore, there was no statistical linkage between marine snow composition and the measured environmental parameters nor between marine snow composition and ambient plankton biomasses. However, groups showing relatively low plankton biomass were generally also rare in the particles (<1%). Most taxa present in marine snow samples showed, either over- or under-representation in the particles compared to expectations based on their relative contribution to the overall plankton biomass (Figs 3 and 4 and S2). For instance, Cnidaria and Chaetognaths were over- as well as under-represented relative to their biomasses, respectively.

An Indicator Species analysis^53,54^ was applied to the 18S rRNA gene data to further investigate potential linkages between marine snow components and stations; i.e. to identify OTUs associated with specific stations. Specific OTUs were found for Stations 11, 14, and 33, but it must be emphasized that the analysis did take multi-level combinations of the different stations into account. However, we choose to focus only on the OTUs associated with single stations due to the large variability in marine snow particles recovered from each station. Four out of the six OTUs associated with Station 11 were similar to dinoflagellates, and the other two OTUs were similar to an appendicularian and a green algae. One OTU similar to Radiolaria was associated with Station 14, and two OTUs associated with Station 33 were both similar to *Oncaea* spp. (Copepoda); see also Table S6. These identified OTUs were all rare and each accounted for <0.01% of the marine snow 18S rRNA gene reads.

We speculated that the prevalence of specific OTUs on marine snow particles could be linked to other OTUs, reflecting spatial and temporal dynamics in the plankton regulating marine snow composition. A SParse InversE Covariance Estimation for Ecological Association Inference (SPIEC-EASI) network analysis^55^ was, therefore, applied to identify OTU-OTU associations. Eight OTUs came out as “key” OTUs (i.e. ≥19 associations with other OTUs), however, these were taxonomically diverse, representing copepods, dinoflagellates, euphausiids, echinoderms, and fish, and all were rare (each accounting for <0.2% of the total number of reads; Fig. S3; Table S5).

## Discussion

Our analysis of 31 individual marine snow particles indicates that the 18S rRNA gene composition is diverse and its regulation complex; however, some patterns emerge:

Copepods dominated the marine snow sequences and the most prominent taxon was related to *Clausocalanus furcatus*. *Clausocalanus* spp. accounted for 17% of the total copepod biomass (573 mg C m^−2^ across all stations sampled for marine snow) and is common in the Sargasso Sea^56^. Other prominent taxa in marine snow were *Oithona* spp. and *Oncaea* spp. These are known to thrive and graze in the vicinity of marine snow particles^57,58^, and *Oithona* spp. and *Oncaea* spp. accounted on average for 12% and 11%, respectively, of the total copepod biomass at the stations sampled for marine snow. Hence, several taxa with high biomasses were also important constituents of marine snow particles.

Cnidarians were the second most abundant group in the marine snow particles. They mainly belonged to Hydrozoa: Siphonophorae and Trachymedusae. At some stations, siphonophores had a significant biomass contribution representing up to 5% of copepod biomass (Table S1). This is consistent with earlier observations, indicating that dry weight and displacement volumes of siphonophores accounted for 8% and 18%, respectively, of the total net zooplankton in the upper 500 m of the Sargasso Sea^59^. The contribution of appendicularians to marine snow particles was very low, which is in accordance with their overall very low densities (Table S1). It is, however, conceivable that our sequencing based approach has underestimated the contribution of gelatinous plankton to the marine snow composition due to their low carbon content; e.g. for appendicularian houses^60^.

Radiolarians were widespread in the marine snow particles and present at all stations, though at very low densities at some stations (Fig. 3, Table S1). They accounted for 10 of the 50 most abundant OTUs, and of these, six were related to Acantharia (Table S2). Large radiolarians (av. size 5 mm) occasionally showed biomasses more than 100–1000 times higher than that of small radiolarians (av. size 0.15 mm) and were equal to copepod biomass or even exceeded copepod biomass at three stations. Rhizaria, which includes Radiolaria and Phaeodaria, was recently estimated to account for 5% of the total oceanic biota carbon reservoir in the top 200 m, which is equivalent to that of all other mesozooplankton in oligotrophic intertropical open oceans^61^. Moreover, radiolarians, especially collodarians, were estimated to be strongly associated with carbon fluxes in oligotrophic^62^ as well as productive^63^ systems. Our data indicate that radiolarians also contribute significantly to marine snow particles and that their contribution to particles can be over-represented compared to their presence in plankton samples irrespective of the fact that we analysed plankton samples alive and assured gentle handling.

Alveolates, consisting mostly of dinoflagellates, were widespread in the marine snow samples and dinoflagellate and ciliate species were also fairly abundant in the plankton (8 to 42% of the total eukaryotic biomass at the marine snow-sampled stations, 0–200 m; Fig. 3). Interestingly, three of the seven dinoflagellate OTUs (an unknown symbiont, *Gymnoxanthella radiolariae*, and an uncultured Duboscquella; Table S2) were likely to be either parasitic or symbiotic and were in fact more abundant than non-symbiotic dinoflagellates (59% of the total dinoflagellate reads). Indeed, a recent global circumnavigation study reported sequences of parasites to be widely distributed in the plankton in the euphotic zone of the oligotrophic oceans^62^. Hence, it appeared that dinoflagellates contributed significantly to marine snow composition. It should, however, be noted that since dinoflagellates have large genomes^64^, and the number of 18S rRNA gene copies in microbial eukaryotes correlate with genome size^65^, they generally have more copies of the 18S rRNA gene than smaller flagellates^47,66^. Consequently, the contribution by dinoflagellates to marine snow composition could have been overestimated by our sequencing approach.

As the components of marine snow originate from live and dead plankton organisms in the environment, it would be expected that marine snow composition would mirror the relative biomass of the individual components of the plankton community, even when considering the relatively low number of marine snow particles examined. However, the composition of marine snow only correlated with stations, and at the different stations particle composition would vary (Fig. 5). Multiple factors may affect the composition of marine snow particles and explain the lack of a statistical coupling to the environmental parameters in our study:

The composition of marine snow is likely not only a function of local particle abundance and particle encounter rates, but also how easily components can be incorporated into larger aggregates^67^; i.e. physiological factors may affect the predominance of taxa in marine snow particles. For instance, stickiness may promote association with marine snow, as has been shown for diatoms^24^. This could contribute to the high occurrence of radiolarians in our marine snow samples, since colonial forms of Polycystinea are known to be gelatinous in nature^68^. Similarly, this could be the case for Cnidaria that appeared over-represented in marine snow relative to their biomass proportion in the plankton (Fig. S2). However, due to the low carbon biomass of Cnidaria^42^, this could have led to the offset between *in situ* biomass and relative contribution to snow particles. Using wet weight instead of carbon biomass could therefore lead to a different outcome. Moreover, marine snow composition and the resemblance with the plankton community will also be influenced by degradation rates of organic matter components. For instance, the relatively resistant chitin exoskeleton of copepods^69^ could cause an accumulation over time of copepod remains in marine snow compared to other more degradable remains, the influence of which would depend on particle age.

Lateral transport may transfer particles formed by one plankton community to another area dominated by a different plankton community. Especially slow sinking particles would be susceptible to lateral transport^70^. In such cases marine snow composition may not correspond to the composition of the contemporary plankton. Indeed, such a mismatch could also be affected by selective processes removing marine snow particles, such as grazing or microbial degradation^3^.

As mentioned, although 18S rRNA gene metabarcoding as done here may be considered semi-quantitative^52^, differences in gene copy number per genome and in genome sizes between species/groups, or preferential amplification of some DNA templates, have the consequence that sequence composition may not equally represent their relative biomass contributions. This likely led to the lack of any linkage between 18S rRNA gene composition of marine snow particles and biomass contribution of specific groups in the plankton (*sensu* Fig. S2).

## Concluding Remarks

Our molecular assessment revealed that the composition of marine snow particles was highly diverse and variable between particles; nevertheless, the composition was statistically linked to sampling locality. We suggest that a combination of the physical/biological environment, characteristics (e.g. stickiness, durability, and abundance), and behaviour (e.g. filtration, feeding, and fecal pellets production) regulate the prevalence of specific plankton taxa in marine snow particles. The composition of marine snow particles was markedly different than in more eutrophic regions where marine snow particles are often dominated by copepod, diatom, and appendicularian material^10,20^, probably due to the fundamental differences in food web structure and possibly in removal mechanisms, such as particle grazing and microbial degradation. Our study shows that 18S rRNA genes from marine snow particles in the oligotrophic Sargasso Sea was dominated by copepods, hydrozoans, radiolarians, and dinoflagellates, with a rather sporadic contribution by *Trichodesmium* and picocyanobacteria. The extent to which these findings can be generalized for oligotrophic oceans and have implications for local particle densities, and consequently for sinking rates and carbon sequestration, needs further interrogation.

## Methods

The sampling grid was designed based on high resolution satellite sea surface temperature observations (Fig. 1) obtained from the GODAE High Resolution SST – pilot project (http://ghrsst-pp.org) including data from the satellites: ENVISAT, NOAA 17 and 18, MODIS Aqua, and AMSR-E. Observations were merged into a 0.05 by 0.05 degrees spatial grid using optimal interpolation that accounts for the statistical properties of the observations and individual noise and bias levels^71^. Only night-time data were used.

Vertical profiles of salinity, fluorescence, and temperature were measured from 0–400 m using a Seabird 9/11 CTD equipped with a 12 Niskin bottle (30 L) rosette sampler (Fig. 1). Fluorescence was converted to chlorophyll *a* (chl *a*) using the relationship between fluorescence and measured chl *a* from the same area and time point from a previous investigation^39,72^. An Underwater Vision Profiler^73^ was used to gain *in situ* abundances of marine snow particles from 0–400 m. Images were acquired and analysed in real time every 20 cm at a 1 m s^−2^ lowering speed. Each image had a recorded volume of 0.93 L. Pixel area was converted to size (mm^2^) using S_m_ = 0.0032 S_p_^1.36^, with S_p_ being the surface of the particle in pixels and S_m_ the surface in mm^2^. Pictures of particles with size >30 pixels (~500 µm) were also individually extracted for taxonomic identification. Images were classified under the EcoTaxa online collaborative software to different taxonomical or morphological categories^74^. All images are available in the following project: http://ecotaxa.obs-vlfr.fr/prj/22.

### Sampling and analysis of plankton

#### Mesozooplankton

Mesozooplankton samples were collected for composition and biomass analysis, and individual specimens were collected for the generation of an 18S rRNA gene reference database of predominant mesozooplankton taxa. Samples were obtained using a multiple opening and closing net (Multinet HydroBios, 0.25 m^2^ mouth opening) with 335 µm meshed nets, towed horizontally (50 to 300 m^3^ net^−1^), and 45 µm meshed nets deployed vertically (10 to 30 m^3^ net^−1^). The large meshed net was employed to sample gelatinous zooplankton (Li *et al*. in preparation), while the small meshed net was used to representatively sample the small sized zooplankton community and abundant gelatinous taxa. Depth discrete samples consistently covered the intervals 0–50 m, 50–100 m, 100–200 m, 200–300, and 300–400 m. At 13 stations, larger sized and patchily distributed zooplankton was also quantitatively sampled using oblique tows from 0–200 m with a 560 µm meshed MIK net (3.5 m diameter ring net) with processed water volumes of >10,000 m^3^ net^−1^. Upon retrieval, samples were stored on ice in thermo-insulated boxes, and selected MIK (560 µm) and Multinet (335 µm) samples (Fig. 1), were analysed alive for rare, large, and non-preservable taxa on a dark field LED light table within 15–30 min (max. 60 min) and 1.5 h after catch for MIK and Multinet samples, respectively. Analyses consisted of species identification, size measurement, and −80 °C freezing of representative taxa and specimens for DNA analysis. After life-sorting, samples were preserved in 4% borax buffered formaldehyde solution for all but MIK net samples, where only 10% of the total volume was preserved for abundant gelatinous zooplankton counts, while the remaining sample was stored in ethanol for fish larvae analyses. All handling of animals was carried out in accordance with relevant guidelines and regulations, as approved by the University of Copenhagen.

Counts of mesozooplankton taxa from the Multinets (copepods, large and small radiolarians, siphonophores, hydromedusae, and appendicularians) and MIK nets (chaetognaths and pelagic tunicates (pyrosomes, salps, and doliolids)) were converted into biomass using published carbon regressions (Table S3). Depth integrated biomass was used to compare marine snow particles to the zooplankton community composition. MIK biomass data covered the 0–200 m depth stratum only. For comparisons between marine snow and plankton biomass composition, Multinet data were depth integrated to the maximum depth at which the marine snow particle was obtained. Particles caught within the mixed layer were assumed to have been exposed to the entire biomass of the plankton community from the surface to the bottom of the mixed layer. The depth of the mixed layer was determined based on a temperature change relative to the surface temperature of 0.5 °C and a sigma-t (density) change relative to the surface of 0.125^75^. If a marine snow particle was caught within the mixed layer, the plankton biomasses for the marine snow capture depth intervals were depth integrated to the mixed layer depth. Biomass was estimated based on published regressions (Table S3) and depth integrated by trapezoid integration.

Marine snow aggregates were collected at 8 stations (Fig. 1) using a custom made 90 µm “Appinet” with a 1 m mouth diameter. The 1.5 m long net ends in a canvas bag holding a large plexi glass cod end (diameter: 30 cm, height: 46 cm) with a volume of 32.5 L^76^. The construction was designed to minimize damage to fragile planktonic organisms and marine snow aggregates. Sampling was done with vertical tows from the sub-surface fluorescence max. (approx. 110–150 m depth) to the surface with a speed of 0.1 m s^−1^. After retrieval, the canvas bag holding the transparent cod end was zipped off, gently carried to the lab, and immediately analyzed for marine snow particles on a light table. Undisturbed marine snow aggregates, not considering *Trichodesmium* puffs, were individually removed using a glass pipette with a suction tube, rinsed with 0.2 µm filtered seawater, photographed if possible due to ship vibration, and stored in Eppendorf tubes at −80 °C until DNA extraction (Table S4). Sizes were estimated to 1–10 mm (length).

#### Picoplankton, phytoplankton, and microzooplankton

Samples for enumeration of *Synechococcus*, *Prochlorococcus*, and picoeukaryotes were fixed with 0.2 µm filtered glutaraldehyde (Sigma; 1% final concentration) and stored at −80 °C. Cells were enumerated using flow cytometry (FACS Calibur, Becton Dickinson) following staining with SYBR Green I^77^ (Molecular Probes). Abundances were converted to biomass using published carbon conversion factors (Table S3) and cell sizes previously obtained from the area^39^. Biomass data were depth integrated by trapezoid integration assuming even distribution.

Samples for enumeration of ciliates and heterotrophic dinoflagellates were fixed with acid Lugol’s solution (2% final concentration) and stored in the dark at 5 °C. Protozoans settled for 24 h in 50 ml sedimentation chambers and were enumerated and sized under an inverted microscope^78^. Cells were identified to the lowest possible taxonomical level, and groups were subdivided into 10 μm equivalent spherical diameter intervals. Volumes were estimated by means of appropriate morphology-derived volume relationship equations. An earlier study^79^ argued that heterotrophy can be assigned to athecate dinoflagellates of unknown trophy, thus all athecate dinoflagellates of unknown trophy were considered heterotrophic. Estimations of cellular carbon content was done using a generic zooplankton volume:carbon conversion factor (Table S3) and depth integrated by trapezoid integration assuming even distribution.

#### Molecular analyses

Database incompleteness is a main limitation of metabarcoding, which can be partially overcome by obtaining sequences from identified local plankton specimens^48,49^. A custom made DNA library of the most prominent plankton taxa was, therefore, constructed with a total of 75 individually sequenced specimens representing the putative main zooplankton groups (based on morphological identification) for comparison with the marine snow sequences (Fig. S1). DNA was extracted (E.Z.N.A. Tissue DNA Kit), quantified (Quant-IT PicoGreen, Invitrogen), and the V7 region of the 18S rRNA genes was PCR amplified using the universal primers UnivF-1183mod and UnivR-1443mod^80^ and MyTaq Red DNA Polymerase (Saveen & Werner). Thermal conditions were 94 °C for 2 min, followed by 35 cycles of 94 °C for 30 s, 49 °C for 30 s, and 68 °C for 45 s, and a final elongation at 72 °C for 5 min. PCR products were purified (E.Z.N.A. Cycle Pure Kit) and Sanger sequenced (Eurofins, Germany). Sequences were assigned taxonomy using the Basic Local Alignment Search Tool^81^ (BLASTN). Top matches and identities were compared to the morphological identifications of the sequenced specimens. In cases where nearest relatives included several species with equal similarities, taxonomy was assigned using the lowest possible common taxonomic denominator. For final taxonomy evaluation of the database, a neighbour-joining tree (bootstrap = 2000) was constructed in MEGA6^82^ using the custom-made plankton database and sequences of nearest relatives from GenBank.

For marine snow particle analyses, DNA was obtained using phenol-chloroform extraction^83^ and 18S rRNA genes were PCR amplified as above but with primers indexed for Illumina sequencing. PCR reactions were done in triplicates and pooled before purification (Agencourt AMPure XP magnetic bead system, Beckman Coulter Life Sciences) and quantification (PicoGreen). Samples were pooled at equimolar concentrations and submitted for paired end sequencing on an Illumina MiSeq V2 2 × 250 nt (National High-throughput DNA sequencing Centre, University of Copenhagen, Denmark). The 18S rRNA amplicon reads were assembled and trimmed to a median length of 224 nucleotides and de-multiplexed with a phred score of Q30 using QIIME v1.9^84^. Removal of singletons and OTU (Operational Taxonomic Units) clustering at 99% similarity was done in USEARCH v8.1.1756^85^ using the UPARSE-OTU algorithm^86^ with implicit chimera check. Taxonomy assignment for the OTUs obtained from the marine snow particles were first attempted using the RDP classifier^87^ (the Ribosomal Database Project) as implemented in QIIME1. However, the RDP confidence score suggested a high number of novel sequences in poor agreement with the current databases at the time. Therefore, we proceeded with manually blasting OTU sequences of interest (see also Tables S2, S5 and S6) using BLASTN^81^, which was validated against our custom-made plankton database, see Fig. S1.

Data on cyanobacteria in the marine snow particles were obtained from an associated study^88^. Briefly, 16S rRNA genes were PCR amplified using the universal primers 341 F and 806R^89^ and Illumina sequenced. All reads were merged, quality- and length trimmed, OTU-clustered on a 97% identity level using the CLC Genomics Workbench 8.0.3 (QIAGEN, Aarhus, Denmark), and assigned taxonomy using the ARB-SILVA database.

#### Statistical and network analyses

The analyses were carried out in R version 3.4.4^90^ and RStudio version 1.1.447^91^, see also supplementary data of the Rmarkdown for the analyses performed in this study. The marine snow particle composition was visualized using a principal component analysis (PCA) in the DESeq. 2 package^92^. A Generalized Linear Model (GLM) analysis, using mvabund^93^, was applied to the 18S rRNA gene OTU abundance table to investigate the composition in relation to station, plankton biomass, and to test for potential environmental drivers (i.e. salinity, temperature, depth, particle abundance, and chl a) of marine snow composition. Furthermore, an indicator species analysis (indicspecies^53^) and a SParse InversE Covariance Estimation for Ecological Association Inference (SPIEC-EASI) network analysis^55^ were used to identify OTUs associated with specific stations and co-variance (potential linkage) between OTUs, respectively. The Indicator analysis was performed on OTUs accounting for >1% of the reads across the dataset, with abundances transformed to proportion. In order to avoid potential problems of unbalanced sampling groups equalized indices were used^53,54,94^. These add equal weights to all site groups, therefore assuming that all have the same ecological variability^54^. The SPIEC-EASI network analysis output was visualized in Cytoscape 3.6.1^95^.

## Supplementary information

Supplementary Dataset 1

Supplementary Dataset 2

Supplementary Dataset 3

Supplementary Dataset 4

Supplementary Dataset 5

Supplementary Dataset 6

Supplementary information

## Data Availability

Nucleotide sequences for the 18S rRNA gene analyses of marine snow aggregates and the plankton community database have been deposited in GenBank, National Center for Biotechnology Information, under accession numbers SRR6157677 and KY594837-KY594911, respectively. All images from the UVP with their identifications, along with selected images of sequenced marine snow particles, have been deposited on EcoTaxa under the project “UVP5 Sargasso 2014” at http://ecotaxa.obs-vlfr.fr/prj/22 and https://ecotaxa.obs-vlfr.fr/prj/2196, respectively.
