# Supplementary material for: Eukaryotic and cyanobacterial communities associated with marine snow particles in the oligotrophic Sargasso Sea
Source: Sci Rep. 2019 Jun 20;9:8891. doi: 10.1038/s41598-019-45146-7 (PMC6586830; doi:10.1038/s41598-019-45146-7)
Supplement: Supplementary file 1 — Supplementary Dataset 1 [file 41598_2019_45146_MOESM1_ESM.pdf]

SUPPLEMENTARY DATA 1

Eukaryotic and cyanobacterial communities associated to marine snow particles in the oligotrophic Sargasso Sea

Regitze B. C. Lundgreen, Cornelia Jaspers, Sachia J. Traving, Daniel J. Ayala, Fabien Lombard, Hans-Peter Grossart, Torkel G. Nielsen, Peter Munk, Lasse Riemann

Below is a list of the files needed for running the scripts in the present document. The files needed are provided online as supplementary data with the paper by Lundgreen et al.:

- Supplementary data 2_MarineSnow_OTUs (.csv file)
  Supplementary data AND needed if you want to run the code from the M_Snow_documentation

- Supplementary data 3_MarineSnow_OTUs_1perc_proportions (.csv file)
  Supplementary data AND needed if you want to run the code from the M_Snow_documentation

- Supplementary data 4_MarineSnow_OTUs_ranking (.csv file)
  Supplementary data AND needed if you want to run the code from the M_Snow_documentation

- Supplementary data 5_Regitze_snow_OTUs_w_biomassdata_use (.csv file)
  Supplementary data AND needed if you want to run the code from the M_Snow_documentation

- Supplementary data 6_Sargasso_Sea_mapping_file (.csv file)
  Supplementary data needed if you want to demultiplex raw fastq file

Rmarkdown for the manuscript “Eukaryotic and cyanobacterial communities associated to marine snow particles in the oligotrophic Sargasso Sea”

January 2018, modified May 2019

Data

18S sequence data clustered into operational taxonomic units (OTUs) using 99% similarity. Sequences were obtained from marine snow particles collected in the Sargasso Sea as described in Lundgreen et al. The abundances were transformed to proportions for the Indicator analysis, and only OTUs with a total abundance of > 1% across the dataset was analyzed.

```
Snow <- read.csv("MarineSnow_OTUs.csv", header = TRUE)
Snow.ab <- read.csv("MarineSnow_OTUs_1perc_proportions.csv", header = TRUE)
```

Libraries

```
library(MASS)
library(survival)
library(readr)
library(fitdistrplus)
library(mvabund)
library(tidyverse)
library(lubridate)
library(permute)
library(indicspecies)
library(lattice)
library(vegan)
library(ggplot2)
```

Check of data distribution

Normal distribution

```
snow.all<-as.vector(as.matrix(t(Snow[,10:834]))) #for data distribution check
fitn <- fitdist(snow.all,"norm")
plot(fitn)
```

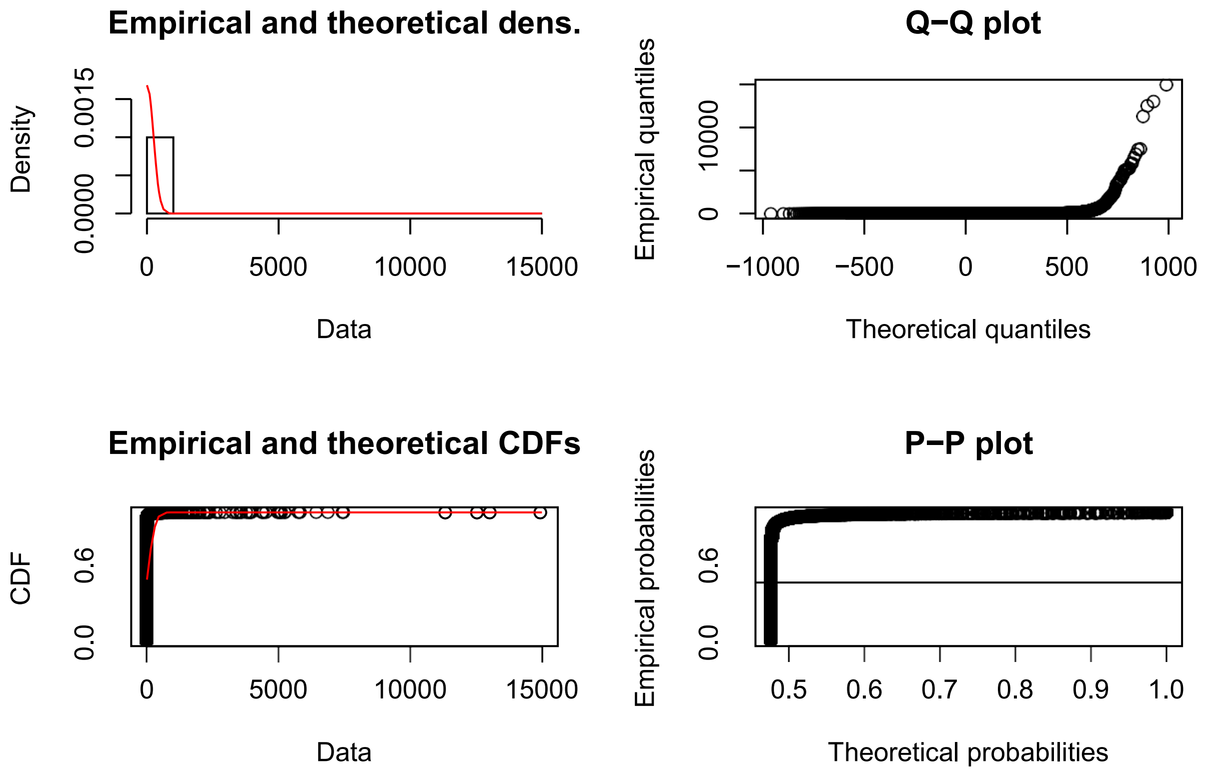

Negative binomial

```
fitnb<-fitdist(snow.all,"nbinom")
plot(fitnb)
```

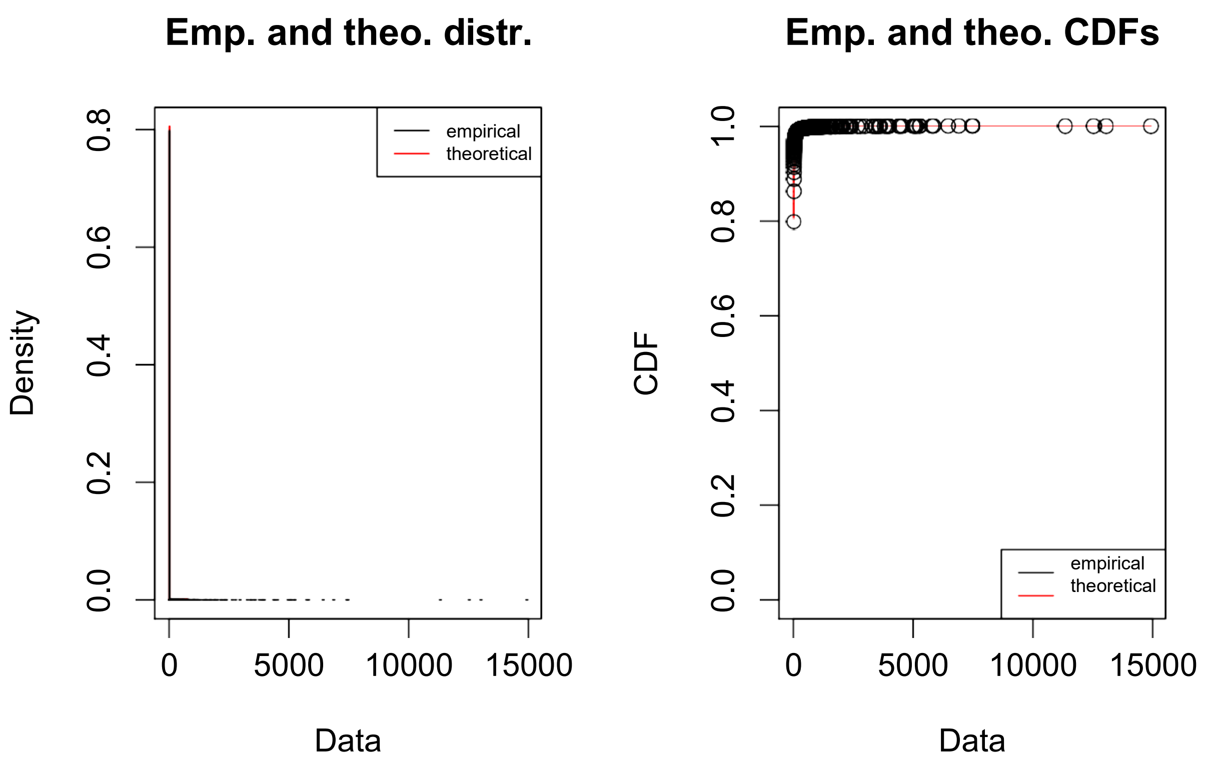

Poisson

```
fitp<-fitdist(snow.all,"pois")
plot(fitp)
```

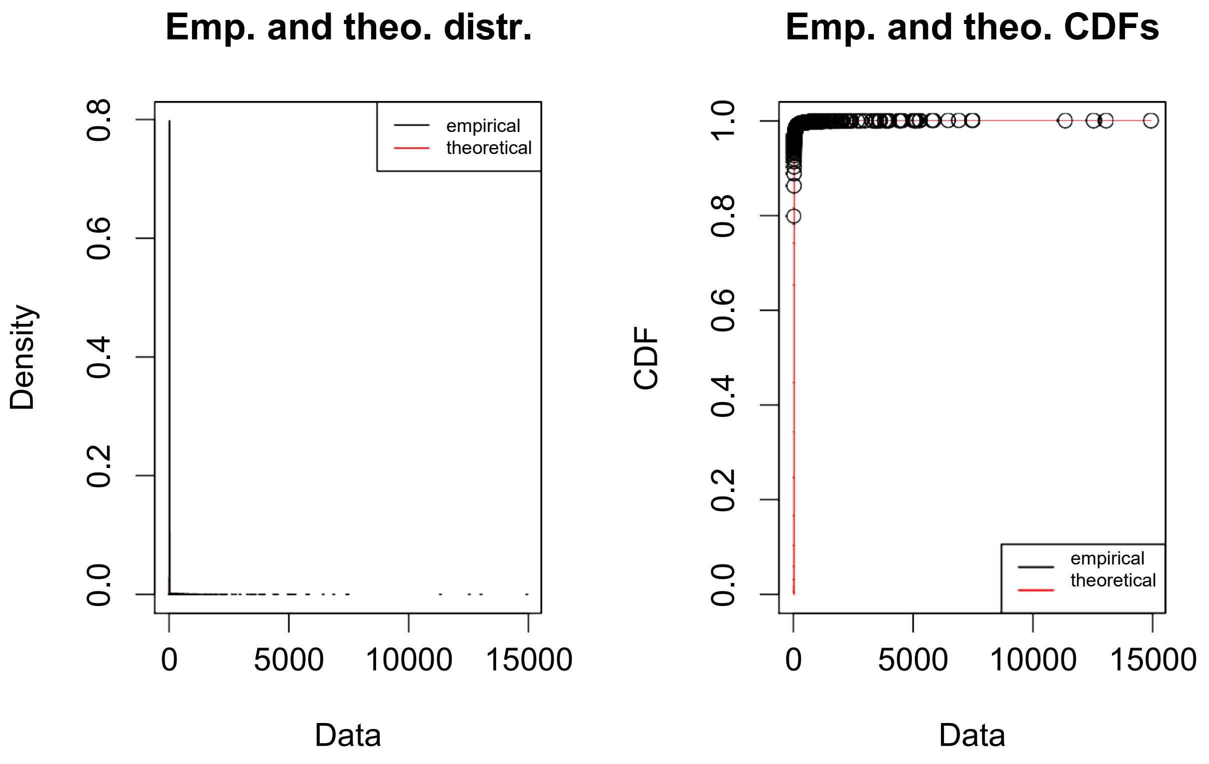

Best fit is a negative binomial distribution.

Generalized Linear Models

Using mvabund by Wang et al., (2012) https://doi.org/10.1111/j.2041-210X.2012.00190.x

```
attach(Snow) #remember to detach when done
snomvabund<-mvabund(Snow[,10:834]) #only OTUs
```

Mean-variance relationship

For a negative binomial the relationship should be quadratic.

```
meanvar.plot(snomvabund,col=as.numeric(station))
```

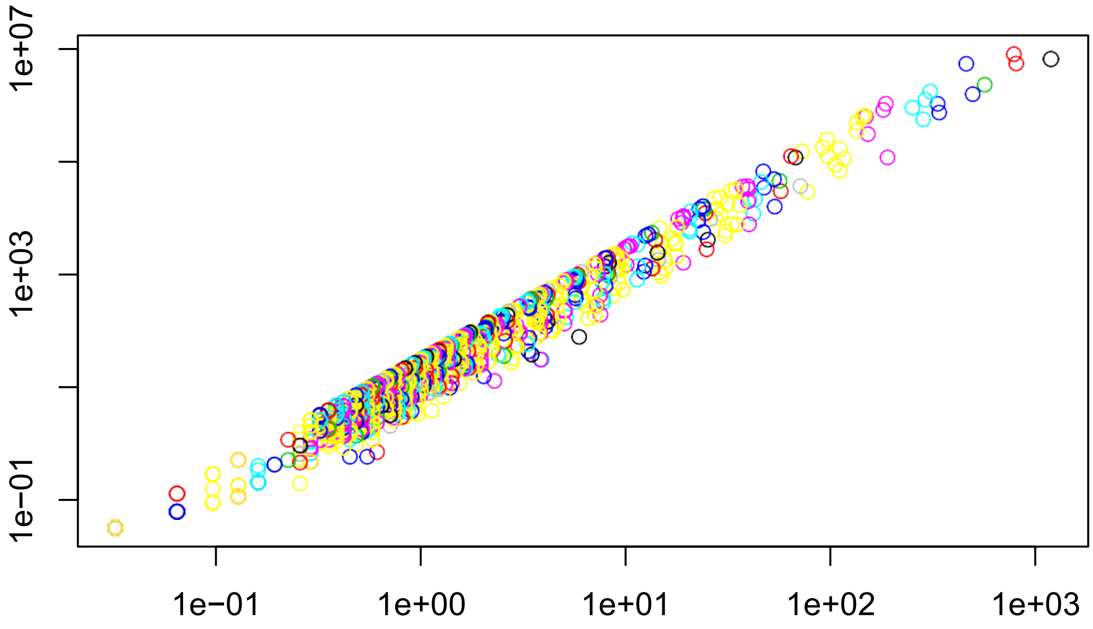


```
detach(Snow)
```

New Analyses of data

Performed 26. March 2018.

To check any relationships between OTUs and plankton biomass, a GLM was produced using the plankton biomasses as the predictor variables and the OTUs as the response variables.

```
data <- read_csv(file = "Regitze_snow_OTUs_w_biomassdata_use.csv", col_names = TRUE,
    na = c("", "NA", "NAN", "ND"))
```

```
## Parsed with column specification:
## cols(
##   .default = col_double(),
##   station = col_character(),
##   Pyrosomatida = col_character()
## )
```

```
## See spec(...) for full column specifications.
```

```r
dat <- gather(data, "Plankton.grps", "biomass", 2:17)
attach(dat)
otus <- mvabund(dat[, 2:239])  #OTUs
tas.st <- manyglm(otus ~ Plankton.grps, family = "negative.binomial")
plot(tas.st)
```

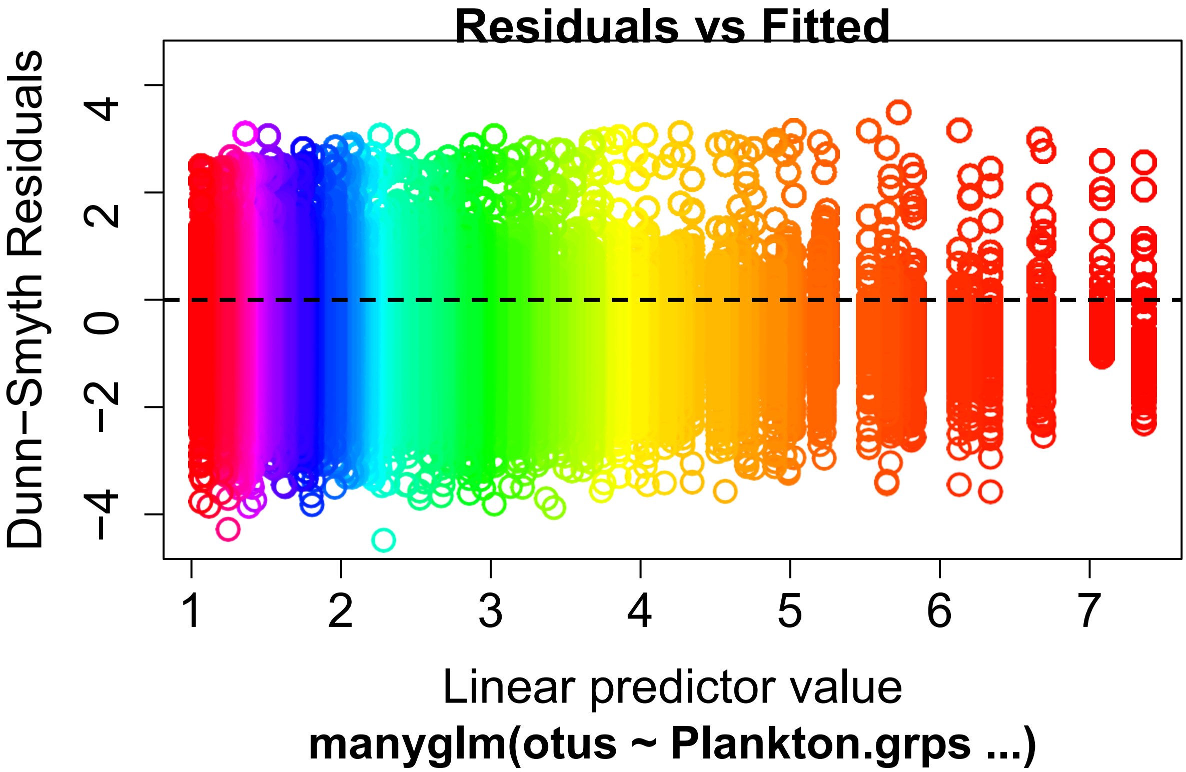


```r
detach(dat)
```

```r
glm.st<-anova(tas.st,p.uni="adjusted",resamp="perm.resid", nBoot = 999, test="LR")
capture.output(anova(tas.st,p.uni="adjusted",resamp="perm.resid", nBoot = 999, test="LR"),file="anovare
st.p<-glm.st$uni.p
st.test<-glm.st$uni.test
write.table(st.p,file("Snow_station_GLM_p.csv"),sep=",")
write.table(st.test,file("Snow_station_GLM_test.csv"),sep=",")
st.table<-glm.st$table
write.table(st.table,file("Snow_plankton_anova_999.csv"),sep=",")
```

Additional Analyses

Indicator Species

1. May 2018 This type of analysis will allow you to look for "indicator species", or in our case OTUs associated with specific groups. We choose to only test individual stations, not combinations (duleg =TRUE). In order to avoid the potential problem og unbalanced sampling, group-equalized indices should be used (Tichy and Chytry, 2006 https://doi-org.ezproxy.library.ubc.ca/10.1111/j.1654-1103.2006.tb02504.x; De Caceres and Legendre, 2009 https://doi-org.ezproxy.library.ubc.ca/10.1890/08-1823.1).

Group equalizes indices give equal weights to all site groups, therefore assuming that all have the same ecological variability (De Caceres et al., 2010 https://doi-org.ezproxy.library.ubc.ca/10.1111/j. 1600-0706.2010.18334.x). Here we applied group-equalized indices, as we assume that all sites have the same variability, and that any variability in site size is random, not reflecting biological/ecological variability.

```
ind.mat<-Snow.ab[,9:213]
station<-Snow.ab[,2]
set.seed(321)
ind1 = multipatt(ind.mat,station,func="IndVal.g", control=how(nperm=99))
summary(ind1,indvald=TRUE)
```

```
##
##  Multilevel pattern analysis
##  ---------------------------
##
##  Association function: IndVal.g
##  Significance level (alpha): 0.05
##
##  Total number of species: 205
##  Selected number of species: 21
##  Number of species associated to 1 group: 9
##  Number of species associated to 2 groups: 5
##  Number of species associated to 3 groups: 2
##  Number of species associated to 4 groups: 1
##  Number of species associated to 5 groups: 3
##  Number of species associated to 6 groups: 1
##  Number of species associated to 7 groups: 0
##
##  List of species associated to each combination:
##
##  Group st11  #sps.  6
##          stat p.value
## OTU_151 0.999 0.03 *
## OTU_113 0.998    0.03 *
## OTU_67  0.984    0.04 *
## OTU_132 0.953    0.03 *
## OTU_153 0.950    0.04 *
## OTU_127 0.949    0.04 *
##
##  Group st14  #sps.  1
##          stat p.value
## OTU_133 0.963 0.02   *
##
##  Group st33  #sps.  2
##         stat  p.value
## OTU_53 0.991    0.05 *
## OTU_37 0.985    0.05 *
##
##  Group st11+st14  #sps.  2
##          stat p.value
## OTU_180 0.973    0.05  *
## OTU_200 0.956    0.03 *
##
##  Group st11+st33  #sps.  2
```

```
##          stat p.value
## OTU_49  0.996    0.01 **
## OTU_161 0.949    0.04 *
##
##  Group st14+st33  #sps.  1
##          stat p.value
## OTU_204 0.933    0.04 *
##
##  Group st11+st12+st14  #sps.  1
##          stat p.value
## OTU_378 0.912    0.05 *
##
##  Group st11+st14+st33  #sps.  1
##         stat p.value
## OTU_45 0.992    0.01 **
##
##  Group st12+st14+st28+st30  #sps.  1
##           stat p.value
## OTU_1255 0.992    0.03 *
##
##  Group st11+st12+st14+st20+st28  #sps.  1
##         stat p.value
## OTU_78 0.985    0.02 *
##
##  Group st11+st12+st14+st28+st30  #sps.  1
##        stat p.value
## OTU_7 0.988    0.03 *
##
##  Group st11+st14+st20+st28+st30  #sps.  1
##         stat p.value
## OTU_20 0.996    0.02 *
##
##  Group st11+st12+st14+st20+st28+st30  #sps.  1
##           stat p.value
## OTU_1805 0.996    0.05 *
## ---
## Signif. codes:  0 '***' 0.001 '**' 0.01 '*' 0.05 '.' 0.1 ' ' 1
```

```
indic.ms <- capture.output(summary(ind1, indvalcomp = TRUE))
write.table(indic.ms,file("IndicatorAnalysis_MarineSnow.txt"))
```

Network analysis using SPIEC-EASI

SParse InversE Covariance Estimation for Ecological Association Inference. Install following this guide: http://psbweb05.psb.ugent.be/conet/microbialnetworks/spieceasi.php

```
library(SpiecEasi)
library(phyloseq)
library(seqtime)
```

The following steps is to get the Snow 18S OTU data “into” phyloseq including the info data.

```
Snow1<-read.csv("MarineSnow_OTUs_ranking.csv", header=TRUE)
otu1=otu_table(Snow1[,2:32], taxa_are_rows = TRUE)
```

```r
tax1=tax_table(Snow1[,c(1,33:35)])
p.obj=phyloseq(otu1,tax1) #creates phyloseq object
filterobj=filterTaxonMatrix(otu1,minocc=3,keepSum=TRUE, return.filtered.indices=TRUE) #from seqtime pkg
otu.f=filterobj$mat
taxa.f=tax1[setdiff(1:nrow(tax1),filterobj$filtered.indices),]
#Assembly new plyoseq object from the filtered elements
otu2=otu_table(otu.f,taxa_are_rows = TRUE)
tax2=tax_table(taxa.f)
p.obj.f=phyloseq(otu2,tax2)
```

Notice that in the phyloseq object created, the "taxonomy" columns have been renamed to "ta1".."ta4". Also, as is we filter the OTUs to a minimum occurrence of 2. this can be changed.

SPIEC-EASI analysis

```r
spiec.out=spiec.easi(p.obj.f,method="mb",icov.select.params=list(rep.num=20))
spiec.graph=adj2igraph(spiec.out$refit, vertex.attr=list(name=taxa_names(p.obj.f)), rmEmptyNodes = TRUE
plot_network(spiec.graph,
             p.obj.f,
             type="taxa",
             color="ta4",
             label=NULL,
             point_size=2,
             alpha=0.4,
             line_alpha=0.1
             )
```

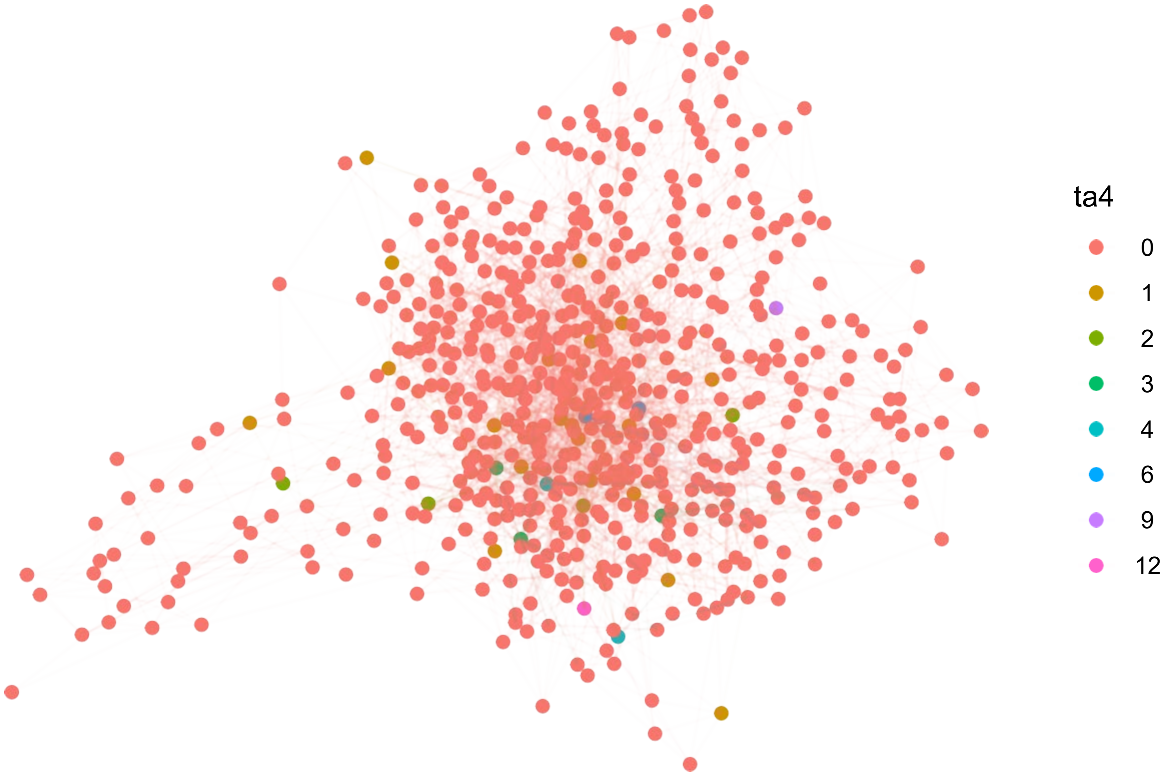

```r
#regression coefficients
betaMat=as.matrix(symBeta(getOptBeta(spiec.out)))
positive=length(betaMat[betaMat>0])/2
negative=length(betaMat[betaMat<0])/2
total=length(betaMat[betaMat!=0])/2
```

```r
#clustering and finding representatives example for membership==1
clusters=cluster_fast_greedy((spiec.graph))
clusterOneIndices=which(clusters$membership==1)
clusterOneOTUs=clusters$names[clusterOneIndices]
```

Export to cytoscape and optimized the visualization of the network.

Export network

Export to cytoscape and optimized the visualization of the network.

```r
>write.graph(spiec.graph,file=file.path("spieceasi_ncol.txt"),format="ncol")
#exporttaxinfo
>write.table(taxa.f,file=file.path("otuname.txt"), sep="\t",quote=FALSE)
edgenum<-spiec.graph$
```

The network and the taxa information was exported as .txt and imported into Cytoscape 3.6.1 and visualized as follows:

Node size = % size, rounded to the nearest whole value*

Color = black

Applied filter color (yellow) = nodes with 19 connections or more. Max connections is 23.

*This way all the very small OTUs are all set to 0 which makes them equally small, since it makes no sense to make the network distinguish between e.g. 0.001 and 0.005

Result summary from Cytoscape

OTUs with the most edges, listed in descending order.

23 edges OTU_109 / sp87

21 edges OTU_96 / sp184

20 edges OTU_415 / sp254

OTU_65 / sp76

19 edges OTU_50 / sp59

OTU_1640 / sp736

OTU_1533 / sp564

Beta-diversity

PCA plots generated for beta-diversity using DESeq2 (Love et al., 2014 doi: 10.1186/s13059-014-0550-8). The plots for the 18S data from this project is described and originally generated for Ayala et al., 2018 DOI:10.1038/s41598-018-24388-x. “‘
