# Supplementary material for: Eukaryotic and cyanobacterial communities associated with marine snow particles in the oligotrophic Sargasso Sea
Source: Sci Rep. 2019 Jun 20;9:8891. doi: 10.1038/s41598-019-45146-7 (PMC6586830; doi:10.1038/s41598-019-45146-7)
Supplement: Supplementary file 7 — Supplementary information [file 41598_2019_45146_MOESM7_ESM.docx]

# **SUPPLEMENTARY MATERIALS**

# **Eukaryotic and cyanobacterial communities associated to marine snow particles in the oligotrophic Sargasso Sea**

Regitze B. C. Lundgreen, Cornelia Jaspers, Sachia J. Traving, Daniel J. Ayala, Fabien Lombard, Hans-Peter Grossart, Torkel G. Nielsen, Peter Munk, Lasse Riemann

**Table S1.** Overview of the biomass (mg C m^-2^) of the dominant plankton groups at the stations sampled for marine snow. All biomasses from 45 µm multiple opening and closing net, if not otherwise indicated and depth integrated to 200 m, which is the greatest marine snow capture depth. ND means no data. For station locations, see Fig. 1.

| **Station** | **11** | **12** | **14** | **18** | **20** | **28** | **30**  **Day** | **30**  **Night** | **33** |
| --- | --- | --- | --- | --- | --- | --- | --- | --- | --- |
| Copepoda | 592 | 211 | 278 | 356 | 364 | 209 | 212 | 342 | 119 |
| Radiolaria* (>1 mm, av. size 5 mm | 0^d^ | 0^d^ | 0^d^ | 0^a,d^ | 0^a,d^ | 2114^d^ | 0^a,d^ | 131^d^ | 122^d^ |
| Radiolaria** (<1 mm, av. size 0.15 mm) | 3 | 0.05 | 0.09 | 0 | 0.27 | 0 | 0 | 0 | 0 |
| Siphonophorae | 23^d^ | ND | 12^d^ | 5^b^ | 3^b^ | 9^d^ | 4^d^ | 15^d^ | 4^d^ |
| Hydromedusae | 0.61^d^ | ND | 0.15^d^ | 0.32^b^ | 0.59^b^ | 0^a^ | 0^a^ | 0.13^d^ | 0^a^ |
| Chaetognatha | 8^b^ | ND | 15 ^b^ | 27 ^b^ | 27 ^b^ | 16 ^b^ | 5^b^ | ND | 21^b^ |
| Appendicularia | 0.60 | 0.29 | 1.31 | 2 | 4 | 0.49 | 0.61 | 0.83 | 0.29 |
| Doliolida | 0.09^b^ | ND | 0.11^b^ | 0.10^b^ | 0.24^b^ | 0.08^b^ | 0.21^b^ | 0^c,b^ | 0.48^b^ |
| Pyrosomatida | 0^b^ | ND | 0^b^ | 0.29^b^ | 0^b^ | 0^c^ | 0.23^b^ | 0.01^b^ | 0.03^b^ |
| Salpida | 0.41^b^ | ND | 0.03^b^ | 0.19^b^ | 0.28^b^ | 0.12^b^ | 0.34^b^ | 0.34^b^ | 1.39^b^ |
| Diatomeae^e^ | 12 | ND | 2 | 1.42 | 3 | 1.46 | 2 | 6 | 3 |
| Ciliophora^e^ | 68 | ND | 30 | 21 | 51 | 58 | 67 | 54 | 86 |
| Dinoflagellata^e^ (Heterotrophic) | 202 | ND | 125 | 64 | 131 | 138 | 130 | 94 | 140 |
| Picoeukaryotes^e^ | 71 | 37 | 52 | 70 | 41 | ND | 50 | 61 | 59 |
| *Prochlorococcus*^e^ | 227 | 159 | 128 | 232 | 137 | ND | 234 | 276 | 245 |
| ***Synechococcus*^e^** | 80 | 74 | 92 | 101 | 118 | ND | 68 | 88 | 76 |

*based on unpreserved material; **based on formalin preserved material; ^a^presence confirmed from non-quantitative MIK net; ^b^abundances from quantitative MIK net; ^c^present in low densities; ^d^abundances from 330 µm multinet; ^e^from depth discrete water samples (see methods section for details).

**Table S2**. Taxonomic affiliations of the top 50 most abundant operational taxonomic units (OTUs) in the sampled marine snow particles. *Nearest relative in BLAST. If no nearest relative is noted, it means taxonomy was solely determined from phylogenetic trees including the custom plankton database due to insufficient online database coverage.

| OTU | Taxonomy | Nearest relative* | Similarity, % | Coverage, % | % of reads | OTU | Taxonomy | Nearest relative* | Similarity, % | Coverage, % | % of reads |
| --- | --- | --- | --- | --- | --- | --- | --- | --- | --- | --- | --- |
| OTU_1805 | Copepoda | *Clausocalanus furcatus* | 98 | 100 | 14.5 | OTU_21 | Copepoda | *Lucicutia ovaliformis* | 99 | 100 | 1 |
| OTU_2 | Hydrozoa | *Abylopsis eschscholtzii* | 100 | 100 | 10.9 | OTU_30 | Radiolaria | *Lychnaspis giltschi* | 100 | 100 | 0.9 |
| OTU_5 | Copepoda | *Paracalanus parvus* | 99 | 100 | 7.4 | OTU_29 | Dinoflagellata | Uncultured dinoflagellate | 96 | 99 | 0.9 |
| OTU_4 | Hydrozoa | *Liriope tetraphylla* | 100 | 100 | 7.2 | OTU_36 | Copepoda | *Undeuchaeta major* | 100 | 100 | 0.8 |
| OTU_7 | Copepoda | *Haloptilus longicornis* | 99 | 100 | 5.2 | OTU_526 | Radiolaria | *Acanthometron* sp. | 99 | 100 | 0.7 |
| OTU_8 | Copepoda | *Calanus sinicus* | 100 | 100 | 4.5 | OTU_1021 | Hydrozoa | *Liriope tetraphylla* | 99 | 100 | 0.7 |
| OTU_6 | Copepoda | *Oithona similis* | 99 | 100 | 4.2 | OTU_3 | Copepoda | *Clausocalanus furcatus* | 100 | 100 | 0.7 |
| OTU_1255 | Copepoda | *Oithonidae sp.* | 99 | 100 | 3.1 | OTU_34 | Mollusca | *Benthonella* sp. | 97 | 100 | 0.6 |
| OTU_10 | Copepoda | *Oncaea* sp. | 98 | 100 | 3.1 | OTU_33 | Mollusca | *Raphitoma linearis* | 95 | 100 | 0.6 |
| OTU_13 | Radiolaria | *Xiphacantha alata* | 99 | 100 | 2.8 | OTU_37 | Copepoda | *Oncaea* sp. | 97 | 100 | 0.5 |
| OTU_9 | Anthozoa | *Hormathia pectinata* | 100 | 100 | 2.7 | OTU_48 | Dinoflagellata | - | - | - | 0.5 |
| OTU_14 | Copepoda | - | - | - | 2.6 | OTU_1240 | Copepoda | *Clausocalanus furcatus* | 99 | 99 | 0.5 |
| OTU_22 | Radiolaria | Uncultured polycystinean | 96 | 99 | 2.3 | OTU_35 | Euphausiacea | *Euphausia mutica* | 100 | 100 | 0.5 |
| OTU_31 | Dinoflagellata | Uncultured symbiont | 100 | 100 | 1.7 | OTU_41 | Radiolaria | *Dorataspis loricata* | 100 | 100 | 0.4 |
| OTU_15 | Ciliophora | *Platyophryides* sp. | 99 | 100 | 1.7 | OTU_38 | Entoprocta | *Loxosomella parguerensis* | 99 | 100 | 0.4 |
| OTU_16 | Fungi | *Cladosporium* sp. | 100 | 100 | 1.7 | OTU_290 | Dinoflagellata | *Neoceratium paradoxides* | 99 | 100 | 0.4 |
| OTU_20 | Chaetognatha | *Pseudosagitta gazellae* | 100 | 100 | 1.4 | OTU_819 | Copepoda | *Clausocalanus furcatus* | 98 | 100 | 0.4 |
| OTU_239 | Copepoda | *Calanus sinicus* | 99 | 100 | 1.4 | OTU_46 | Chaetognatha | *Sagitta setosa* | 100 | 100 | 0.4 |
| OTU_17 | Ciliophora | *Platyophrya spumacola* | 99 | 100 | 1.3 | OTU_61 | Dinoflagellata | Uncultured Duboscquella | 100 | 100 | 0.4 |
| OTU_18 | Radiolaria | *Phyllostaurus siculus* | 100 | 100 | 1.3 | OTU_39 | Radiolaria | *Amphilonche elongata* | 100 | 100 | 0.4 |
| OTU_908 | Radiolaria | *Collosphaera tuberosa* | 89 | 100 | 1.2 | OTU_147 | Radiolaria | Uncultured polycystinean | 96 | 99 | 0.4 |
| OTU_19 | Copepoda | *Cyclopinodes* sp. | 95 | 100 | 1.2 | OTU_11 | Radiolaria | *Collosphaera tuberosa* | 89 | 100 | 0.4 |
| OTU_24 | Thaliacea | *Dolioletta gegenbauri* | 100 | 100 | 1.1 | OTU_45 | Dinoflagellata | *Neoceratium platycorne* | 100 | 100 | 0.4 |
| OTU_26 | Appendicularia | *Oikopleura longicauda* | 100 | 100 | 1.0 | OTU_40 | Copepoda | *Anomalocera patersoni* | 95 | 100 | 0.3 |
| OTU_32 | Dinoflagellata | *Gymnoxanthella radiolariae* | 100 | 100 | 1.0 | OTU_53 | Copepoda | *Oncaea* sp. | 96 | 100 | 0.3 |

**Table S3.** Carbon conversion factors used for the various plankton groups. L = length, DW = dry weight, TL= trunk length, UH = umbrella height, D = diameter, BV = biovolume, NH = nectophore height, ZL = zooid length, PL = prosome length.

| Group | Carbon conversion factor | Reference |
| --- | --- | --- |
| Ctenophora | C (mg) = 0.0048 Total length (mm)^1.775^ | Lavaniegos & Ohman 2007 |
| Salpida* | C (µg) = 1.62 L (mm)^1.93^ | Heron et al. 1988 |
| Doliolida | C (µg) = 0.51 L (mm) ^2.28^ | Lavaniegos & Ohman 2007 |
| Pyrosomatida | DW (mg) = 0.111 L (mm)^1.90^  C (mg) = 11.3% of DW | DW equation given in Andersen and Sardou 1994  C as % of DW given in Gorsky et al. 1988  as cited in Lavaniegos & Ohman 2007 |
| Appendicularia^**^ | log C (µg) = 2.455 log TL (µm)-6.96 | Jaspers et al. 2009 |
| Radiolaria (all) | 0.28mgC mm^-3^ | as cited in Biard et al. 2016 |
| Siphonophorae | C (µg) = 20.47 NH/ZL (mm) ^0.834^ | Lavaniegos & Ohman 2007 |
| Scyphomedusae | C (g) = 1.63 10^-7^ D (mm) ^2.9^ | Hirst & Lucas 1998 |
| Hydromedusae | C (µg) = 1.8885 UH (mm) ^2.619^ | Lavaniegos & Ohman 2007 |
| Copepoda (Nauplii) | mg C = 3.18 × 10^-6^ × L_µm_^3.31^ | Berggreen et al. 1988 |
| *Paracalanus*, *Clausocalanus*, and *Calocalanus* spp. (Copepodites) | ln(µg DW) = 3.25 × ln(L_µm_) - 19.65  C:DW = 0.45 | Chisholm and Roff, 1990 |
| *Oithona* spp. (Copepodites) | log(µg DW) = 3.16 × log(L_mm_) - 8.18  C:DW = 0.45 | Hopcroft et al. 1998 |
| All other Calanoida (Copepodites) | ln(µg DW) = 2.74 × ln(L_µm_) - 16.41  C:DW = 0.45 | Chisholm and Roff, 1990 |
| All other Cyclopoida (incl. *Oncaea* spp.: copepodites) | ln(µg DW) = 1.96 × ln(L_µm_) - 11.64  C:DW = 0.45 | Chisholm and Roff, 1990 |
| Harpacticoida (Copepodites) | ln(µg C) = 1.15 × ln(TL_µm_) - 7.79 | Satapoomin, 1999 |
| Chaetognatha | C (µg) = 0.0956 L(µm)^2.9093^ | Lavaniegos & Ohman 2007 |
| Diatomeae | pg C cell^-1^ = 0.288 volume^0.811^ | Menden-Deuer & Lessard 2000 |
| Ciliophora | 0.12 g C cm^- 3^ | Hansen et al. 1997 |
| Dinoflagellata  (Heterotrophic) | 0.12 g C cm^- 3^ | Hansen et al. 1997 |
| Picoeukaryotes | log (pg cell^-1^) = 0.94 log (μm^3^) - 0.6 | Eppley et al. 1970 |
| *Prochlorococcus* | 325 fg C µm^–3^ | DuRand et al. 2001 |
| *Synechococcus* | 325 fg C µm^–3^ | DuRand et al. 2001 |

*: Based on *Thalia democratica* for both aggregate and solitary forms. This regression compared well with all other regressions available for salps, apart from *Ritterielle retracta* and *Pegea social* which were not observed in the samples. These regressions were, therefore, not considered in the evaluation.

**: Appendicularian biomasses used in this study are based on formaldehyde preserved samples, corrected for shrinkage using a factor of 0.13^16,17^.

**Table S4.** Overview of the 31 marine snow particles analyzed in this study.

| **Station #** | **Latitude** | **Longitude** | **Marine snow #** | **Transect** | **Depth (m)** | **Total # raw reads** |
| --- | --- | --- | --- | --- | --- | --- |
| 11 | 26.15.091 N | 068.29.907 W | 110 | 1 | 0-150 | 16,788 |
| 12 | 25.39.794 N | 068.30.359 W | 1 | 1 | 0-50 | 20,422 |
| 12 | 25.39.794 N | 068.30.359 W | 3 | 1 | 0-50 | 59,250 |
| 14 | 24.59.942 N | 065.29.902 W | 134 | 2 | 0-150 | 8771 |
| 18 | 26.49.320 N | 065.30.236 W | 7 | 2 | 0-100 | 9513 |
| 18 | 26.49.320 N | 065.30.236 W | 8 | 2 | 0-100 | 12,536 |
| 18 | 26.49.320 N | 065.30.236 W | 9 | 2 | 0-100 | 5981 |
| 18 | 26.49.320 N | 065.30.236 W | 10 | 2 | 0-100 | 7158 |
| 20 | 27.50.032 N | 065.29.436 W | 11 | 2 | 0-150 | 14,573 |
| 20 | 27.50.032 N | 065.29.436 W | 12 | 2 | 0-150 | 13,738 |
| 20 | 27.50.032 N | 065.29.436 W | 13 | 2 | 0-150 | 12,004 |
| 20 | 27.50.032 N | 065.29.436 W | 14 | 2 | 0-150 | 695 |
| 28 | 25.00.015 N | 062.29.827 W | 19 | 3 | 0-100 | 10,449 |
| 28 | 25.00.015 N | 062.29.827 W | 20 | 3 | 0-100 | 19,238 |
| 28 | 25.00.015 N | 062.29.827 W | 21 | 3 | 0-100 | 25,977 |
| 28 | 25.00.015 N | 062.29.827 W | 22 | 3 | 0-100 | 15,803 |
| 28 | 25.00.015 N | 062.29.827 W | 188 | 3 | 0-200 | 8463 |
| 30 | 25.39.924 N | 062.45.183 W | 23 | 3 | 0-100 | 20,700 |
| 30 | 25.39.924 N | 062.45.183 W | 24 | 3 | 0-100 | 8887 |
| 30 | 25.39.924 N | 062.45.183 W | 25 | 3 | 0-100 | 9770 |
| 30 | 25.37.735 N | 062.48.035 W | 26 | 3 | 0-100 | 12,322 |
| 30 | 25.37.735 N | 062.48.035 W | 27 | 3 | 100-135 | 3058 |
| 30 | 25.37.735 N | 062.48.035 W | 28 | 3 | 135-180 | 6447 |
| 30 | 25.37.735 N | 062.48.035 W | 31 | 3 | 0-100 | 14,621 |
| 30 | 25.38.316 N | 062.47.833 W | 32 | 3 | 0-25 | 15,926 |
| 30 | 25.38.316 N | 062.47.833 W | 33 | 3 | 25-65 | 208 |
| 30 | 25.38.316 N | 062.47.833 W | 34 | 3 | 65-100 | 12,452 |
| 30 | 25.38.584 N | 062.47.606 W | 35 | 3 | 0-100 | 10,176 |
| 30 | 25.38.584 N | 062.47.606 W | 36 | 3 | 135-180 | 22,594 |
| 30 | 25.37.735 N | 062.48.035 W | 39 | 3 | 0-150 | 9349 |
| 33 | 26.39.998 N | 062.44.967 W | 42 | 3 | 0-160 | 3793 |

**Table S5**. Taxonomic affiliations of the operational taxonomic units (OTUs) with most associations to other OTUs in the SPIEC-EASI analysis (Fig. S3). All of these OTUs were rare (each accounting for <0.2% of the total number of reads). *Nearest relative in BLAST.

| OTU | Taxonomy | Nearest relative* | Similarity, % | Coverage, % |
| --- | --- | --- | --- | --- |
| OTU_50 | Copepoda | *Acrocalanus longicornis* | 100 | 100 |
| OTU_65 | Holothuriidae | *Pearsonothuria graeffei* | 100 | 100 |
| OTU_109 | Dinoflagellata | *Pelagodinium beii* | 100 | 100 |
| OTU_96 | Euphausiacea | *Thysanopoda aequalis* | 100 | 100 |
| OTU_983 | Copepoda | *Oithonidae* sp. | 98 | 100 |
| OTU_415 | Euphausiacea | *Stylocheiron abbreviatum* | 97 | 100 |
| OTU_1533 | Teleostei | *Anguilla reinhardtii* | 97 | 99 |
| OTU_1640 | Copepoda | *Calanus finmarchicus* | 96 | 90 |

**Table S6.** Taxonomy of nearest relative of the operational taxonomic units (OTUs) found associated with a station in the indicator analysis. These OTUs make up < 2% of the total number of reads. *Nearest relative in BLAST.

| OTU | Taxonomy | Nearest relative* | Similarity, % | Coverage, % |
| --- | --- | --- | --- | --- |
| OTU_151 | Viridiplantae | *Halosphaera* sp. | 100 | 100 |
| OTU_113 | Dinoflagellata | *Symbiodinium* sp. | 94 | 99 |
| OTU_67 | Dinoflagellata | Uncultured *Duboscquella* | 97 | 100 |
| OTU_132 | Appendicularia | *Oikopleura labradoriensis* | 99 | 100 |
| OTU_153 | Dinoflagellata | *Parvodinium trawinskii* | 100 | 100 |
| OTU_127 | Dinoflagellata | Uncultured *Duboscquella* | 97 | 100 |
| OTU_133 | Radiolaria | *Hexacontium pachydermum* | 89 | 66 |
| OTU_53 | Copepoda | *Oncaea* sp. | 96 | 100 |
| OTU_37 | Copepoda | *Oncaea* sp. | 97 | 100 |


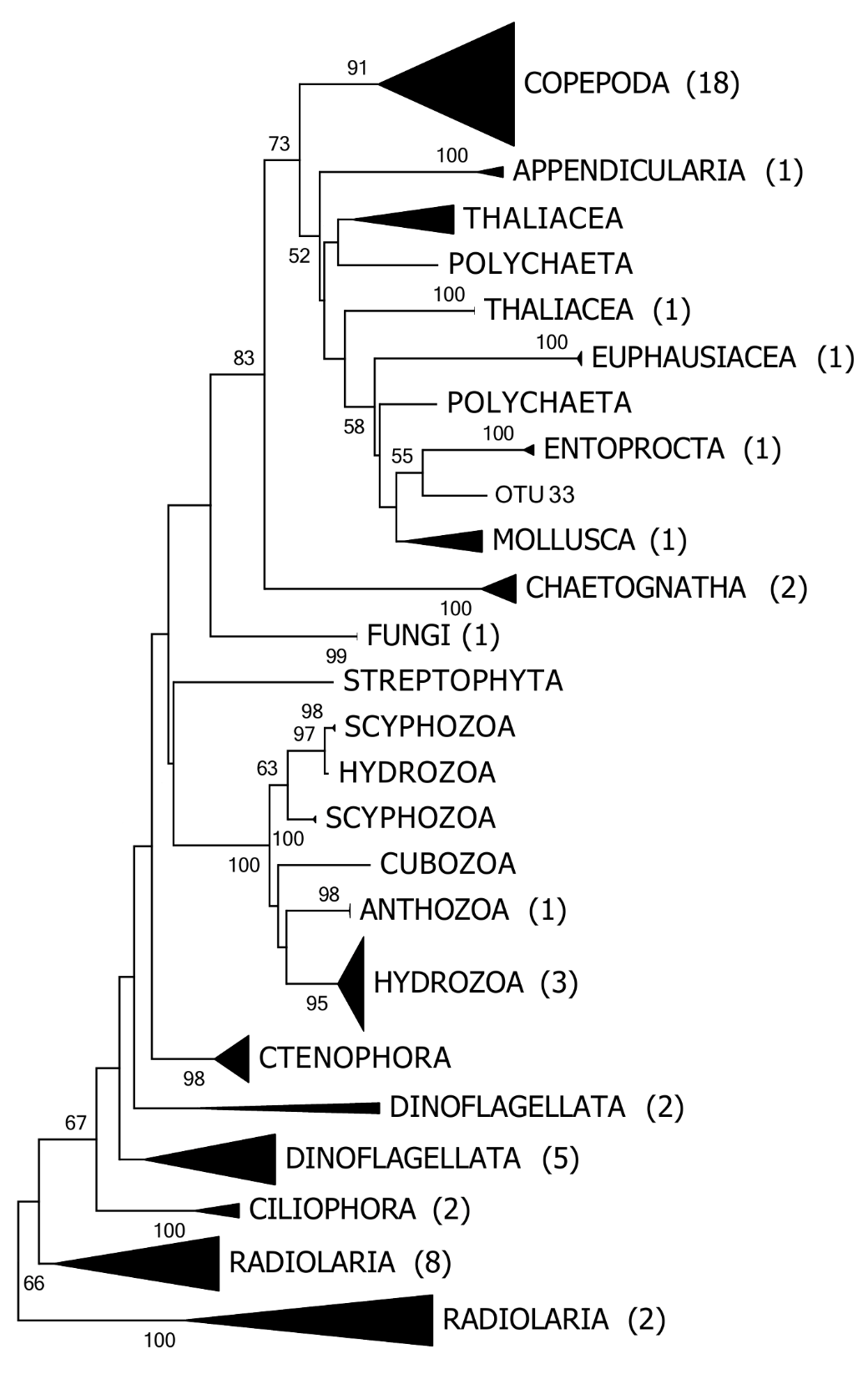


**Fig. S1.** A neighbour-joining tree showing the phylogeny of the 50 most dominant operational taxonomic units (OTUs) obtained from Illumina 18S rRNA gene sequencing of 31 marine snow particles. The tree is built on a total of 173 sequences including those of our custom-made plankton database of 75 morphologically identified plankton organisms, the top 50 most abundant OTUs, and their nearest relatives in GenBank. Groups were identified to lowest possible phylogenetic level. Numbers in parentheses indicate number of OTUs in that group. Bootstrap values greater than 50% (2000 replications) are shown. A group was considered monophyletic if bootstrap values exceeded 50%, and all reference sequences within the group were from the same phylum. Scale bar indicates nucleotide substitutions.


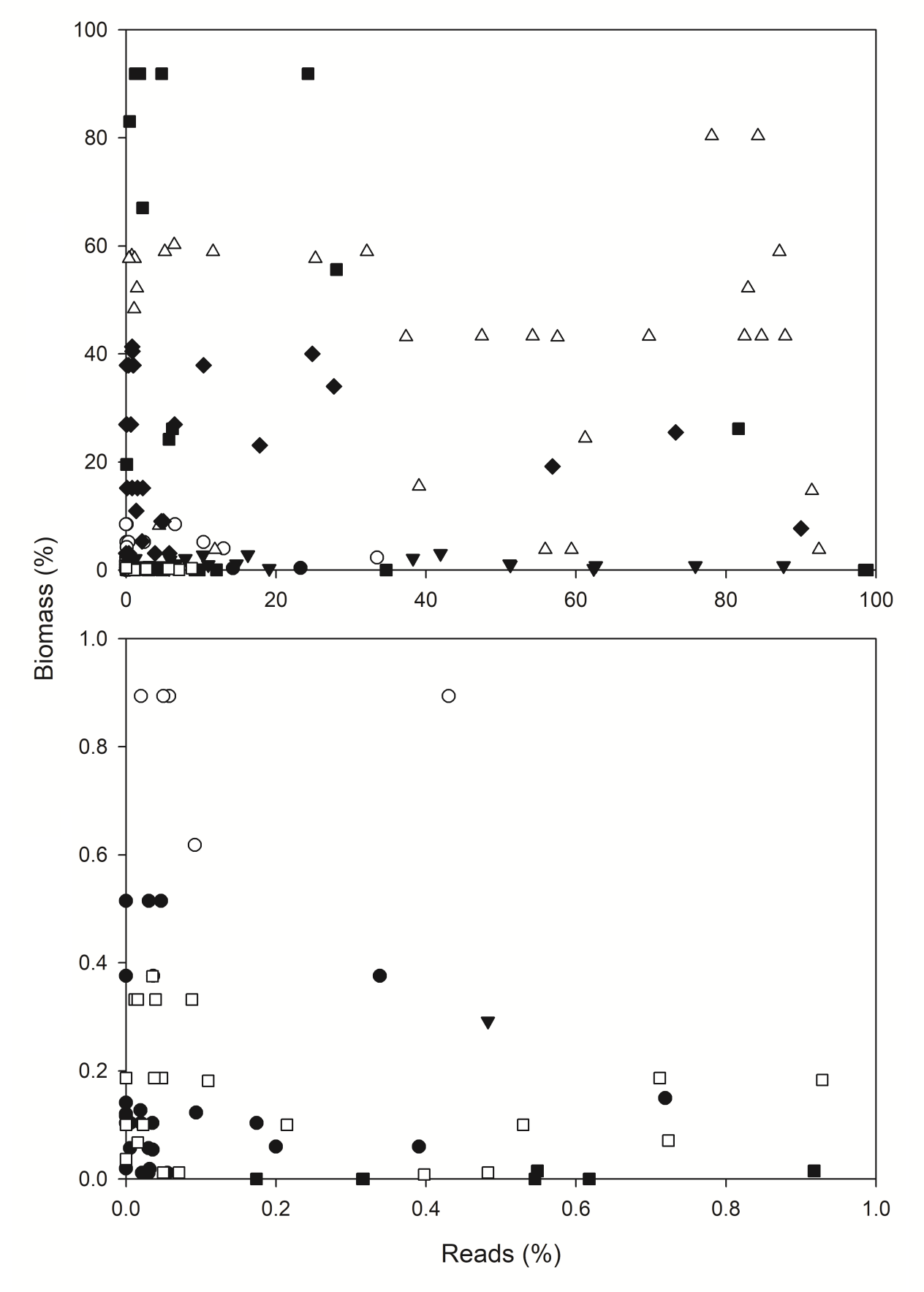


**Fig. S2.** Comparison between the relative proportion of reads of main mesozooplankton taxa in marine snow particles and the relative contribution of these same taxon to plankton biomass within their respective marine snow capture intervals. Note the different scales in a) and b).
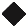
 = Alveolata,
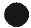
 = Appendicularia,
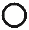
 = Chaetognatha,
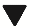
 = Cnidaria,
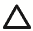
 = Copepoda,
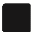
 = Radiolaria,
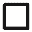
= Thaliacea.

**
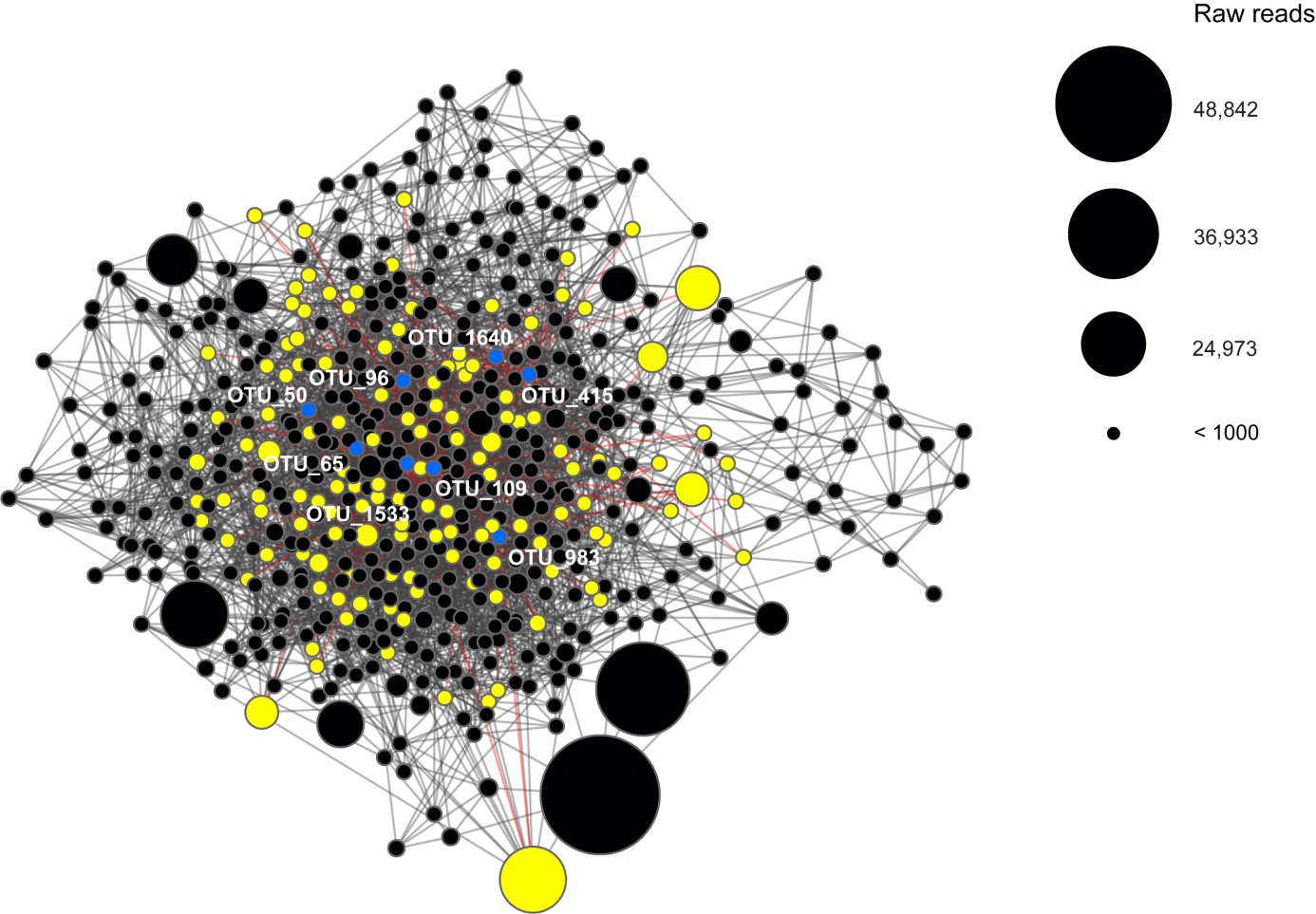
**

**Fig. S3.** Network analysis of 18S rRNA genes from marine snow particles using SPIEC-EASI^18^. Node size is weighted by raw read abundances and blue nodes indicate the operational taxonomic units (OTUs) with **≥**19 edges (maximum number of edges is 23), the OTUs with most associations to other OTUs in the analysis. All of the blue nodes were rare OTUs (each accounting for <0.2% of the total number of reads). Taxonomic affiliations of the OTUs represented by blue nodes are provided in Table S5. Yellow nodes are OTUs directly associated with a blue node, and black nodes are either associated with yellow or other black nodes. Grey and red edges denote positive and negative associations, respectively.

# **SUPPLEMENTARY REFERENCES**

1. Hansen, P. J., Bjørnsen, P. K. & Hansen, B. W. Zooplankton grazing and growth: Scaling within the 2-2,000-µm body size range. *Limnol. Oceanogr.* **42,** 687–704 (1997).

2. Lavaniegos, B. E. & Ohman, M. D. Coherence of long-term variations of zooplankton in two sectors of the California Current System. *Prog. Oceanogr.* **75,** 42–69 (2007).

3. Heron, A., McWilliam, P. & Dal Pont, G. Length-weight relation in the salp *Thalia democratica* and potential of salps as a source of food. *Mar. Ecol. Prog. Ser.* **42,** 125–132 (1988).

4. Andersen, V. & Sardou, J. *Pyrosoma atlanticum* (Tunicata, Thaliacea): Diel migration and vertical distribution as a function of colony size. *J. Plankton Res.* **16,** 337–349 (1994).

5. Gorsky, G. *et al.* C and N composition of some northwestern Mediterranean zooplankton and micronekton species. *J. Exp. Mar. Bio. Ecol.* **124,** 133–144 (1988).

6. Jaspers, C., Nielsen, T. G., Carstensen, J., Hopcroft, R. R. & Møller, E. F. Metazooplankton distribution across the Southern Indian Ocean with emphasis on the role of Larvaceans. *J. Plankton Res.* **31,** 525–540 (2009).

7. Biard, T. *et al.* *In situ* imaging reveals the biomass of giant protists in the global ocean. *Nature* **532,** 504–507 (2016).

8. Hirst, A. G. & Lucas, C. H. Salinity influences body weight quantification in the scyphomedusa *Aurelia aurita*: Important implications for body weight determination in gelatinous zooplankton. *Mar. Ecol. Prog. Ser.* **165,** 259–269 (1998).

9. Berggreen, U., Hansen, B. & Kiørboe, T. Food size spectra, ingestion and growth of the copepod *Acartia tonsa* during development: Implications for determination of copepod production. *Mar. Biol.* **99,** 341–352 (1988).

10. Chisholm, L. A. & Roff, J. C. Size-weight relationships and biomass of tropical neritic copepods off Kingston, Jamaica. *Mar. Biol.* **106,** 71–77 (1990).

11. Hopcroft, R. R., Roff, J. C. & Lombard, D. Production of tropical copepods in Kingston Harbour, Jamaica: The importance of small species. *Mar. Biol.* **130,** 593–604 (1998).

12. Satapoomin, S. Carbon content of some common tropical Andaman Sea copepods. *J. Plankton Res.* **21,** 2117–2123 (1999).

13. Menden-Deuer, S. & Lessard, E. J. Carbon to volume relationships for dinoflagellates, diatoms, and other protist plankton. *Limnol. Oceanogr.* **45,** 569–579 (2000).

14. Eppley, R., Reid, F. & Strickland, J. Estimates of phytoplankton crop size, growth rate, and primary production. *Calif. Univ. Bull Scripps Inst. Ocean.* **17,** 33–42 (1970).

15. Durand, M. D., Olson, R. J. & Chisholm, S. W. Phytoplankton population dynamics at the Bermuda Atlantic Time-series station in the Sargasso Sea. *Deep. Res. Part II Top. Stud. Oceanogr.* **48,** 1983–2003 (2001).

16. Jaspers, C. & Carstensen, J. Effect of acid Lugol solution as preservative on two representative chitineous and gelatinous zooplankton groups. *Limnol. Oceanogr. Methods* **7,** 430–435 (2009).

17. Scheinberg, R. D., Landry, M. R. & Calbet, A. Grazing of two common appendicularians on the natural prey assemblage of a tropical coastal ecosystem. *Mar. Ecol. Prog. Ser.* **294,** 201–212 (2005).

18. Kurtz, Z. D., Müller, C. L., Miraldi, E. R., Littman, D. R., Blaser, M. J., Bonneau, R. A. Sparse and compositionally robust inference of microbial ecological networks. *PLoS Comput. Biol.* **11**, e1004226 (2015).
